# Investigation of the activity of baicalein towards Zika virus

**DOI:** 10.1186/s12906-023-03971-4

**Published:** 2023-05-03

**Authors:** Suteema Sawadpongpan, Janejira Jaratsittisin, Atitaya Hitakarun, Sittiruk Roytrakul, Nitwara Wikan, Duncan R. Smith

**Affiliations:** 1 Institute of Molecular Biosciences, Mahidol University, Salaya, 73170 Thailand; 2 National Center for Genetic Engineering and Biotechnology (BIOTEC), National Science and Technology Development Agency, Pathum Thani, 12120 Thailand; 3 Department of Pharmacology, Faculty of Medicine, Chiang Mai University, Chiang Mai, 50200 Thailand

**Keywords:** Antiviral activity, Baicalein, Zika virus, Half-maximal effective concentration

## Abstract

**Background:**

Zika virus (ZIKV) is a mosquito transmitted virus spread primarily by *Aedes* species mosquitoes that can cause disease in humans, particularly when infection occurs in pregnancy where the virus can have a significant impact on the developing fetus. Despite this, there remains no prophylactic agent or therapeutic treatment for infection. Baicalein is a trihydroxyflavone, that is found in some traditional medicines commonly used in Asia, and has been shown to have several activities including antiviral properties. Importantly, studies have shown baicalein to be safe and well tolerated in humans, increasing its potential utilization.

**Methods:**

This study sought to determine the anti-ZIKV activity of baicalein using a human cell line (A549). Cytotoxicity of baicalein was determined by the MTT assay, and the effect on ZIKV infection determined by treating A549 cells with baicalien at different time points in the infection process. Parameters including level of infection, virus production, viral protein expression and genome copy number were assessed by flow cytometry, plaque assay, western blot and quantitative RT-PCR, respectively.

**Results:**

The results showed that baicalein had a half-maximal cytotoxic concentration (CC_50_) of > 800 µM, and a half-maximal effective concentration (EC_50_) of 124.88 µM. Time-of-addition analysis showed that baicalein had an inhibitory effect on ZIKV infection at the adsorption and post-adsorption stages. Moreover, baicalein also exerted a significant viral inactivation activity on ZIKV (as well as on dengue virus and Japanese encephalitis virus) virions.

**Conclusion:**

Baicalein has now been shown to possess anti-ZIKV activity in a human cell line.

**Supplementary Information:**

The online version contains supplementary material available at 10.1186/s12906-023-03971-4.

## Background

Zika virus (ZIKV), a human pathogenic virus transmitted by *Aedes* (*Ae*.) species mosquitoes is classified as belonging to the genus *Flavivirus*, a part of the family *Flaviviridae*, together with closely related viruses such as dengue virus (DENV), Japanese encephalitis virus (JEV), West Nile virus (WNV), and yellow fever virus (YFV) [1]. ZIKV was first isolated in 1947 from a sentinel rhesus monkey in the Zika forest of Uganda [2]. A year later, the virus was subsequently isolated from a pool of *Ae. africanus* mosquitoes from the same forest [2]. In 1964, ZIKV was isolated from a man who had been resident in Uganda for around two months [3], and this is considered as the first confirmation of ZIKV infection of a human [4]. ZIKV was originally not considered an important human pathogen, because while it circulated within Africa and Asia, only 14 recorded cases of human infection occurred in the 60 years following its first isolation [5]. In 2007 the first outbreak of Zika fever occurred outside of Africa and Asia on the Yap Islands, Federated States of Micronesia [6]. Subsequently, a second outbreak occurred in 2013 in French Polynesia [7]. This outbreak led to ZIKV infection being considered an important public health problem as there were reports showing that ZIKV infection was associated with neurological disorders, especially Guillain-Barré syndrome [8, 9]. A large outbreak of ZIKV infection occurred in Brazil in 2015, and the virus rapidly spread to neighboring countries leading to over a million cases of infection [10–12]. Moreover, a number of cases of congenital microcephaly associated with ZIKV infection were reported for the first time during this outbreak [13, 14].

ZIKV is an enveloped, positive-sense single stranded RNA virus which contains an RNA genome of approximately 10.8 kb in length [15]. The genome of the virus contains one open reading frame (ORF) flanked by 5’ and 3’ untranslated regions. The ORF encodes for 10 viral proteins including three structural proteins (envelope: E, capsid: C, pre-membrane: prM) and 7 non-structural proteins (NS1, NS2A, NS2B, NS3, NS4A, NS2B, NS5) [15]. The ORF is translated into a single polyprotein that is subsequently cleaved by viral and host proteases into the 10 viral proteins. ZIKV has been classified into three lineages, namely the Asian lineage, the West African lineage, and the East African lineage based on phylogenetic analysis [16–18].

Infection with ZIKV primarily occurs by mosquito-to-human transmission, and *Aedes* spp. mosquitoes are the most important vector that transmits the virus to humans [19], but in addition, ZIKV can be transmitted from one person to another by sexual transmission [20–22], blood transfusion [23], and maternal-fetal transmission [24, 25]. Symptoms of ZIKV infection can develop some 3–14 days post-infection [26], albeit that most people who get infected with ZIKV are asymptomatic. Symptoms of ZIKV infection can include headache, fever, myalgia, arthralgia, maculopapular rash, and conjunctivitis [27, 28]. In some cases, ZIKV infection causes complications including GBS in adults and congenital Zika syndrome in developing fetuses. To date, there is no protective vaccine to prevent infection, nor antiviral drug to treat infection.

Baicalein (5,6,7-trihydroxyflavone) is a bioactive compound extracted from plants. It was first isolated from the root of *Scutellaria baicalensis* (Chinese skullcap) [29], but it is also found in *Erigeron breviscapus* [30] and *Oroxylum indicum* (Indian trumpet) [31]. This latter plant (*Oroxylum indicum*) is used in traditional medicines as an anti-inflammatory and antipyretic in a number of Asian countries including Thailand [31]. Baicalein has been demonstrated to have several pharmacological properties including anticancer [32, 33], antioxidant [34, 35], anti-inflammation [36, 37], neuroprotection [38–40], as well as antibacterial [41, 42], and antiviral activities [43–46]. While baicalein has previously been shown to have anti-ZIKV activity [47], that study utilized a non-human cell line (Vero), and this study sought to determine the activity in a human cell line (A549, a human lung carcinoma cell line). This analysis is important, as we have previously shown that another natural compound (kaempferol) showed markedly different antiviral activities in a human (HEK293T/17) and a non-human (BHK-21) cell line [48].

## Methods

### Cells and virus

*Ae. albopictus* C6/36 cells (ATCC: CRL-1660) were cultured in minimum essential medium (MEM; Thermo Fisher Scientific, Waltham, MA) supplemented with 10% heat-inactivated fetal bovine serum (FBS; Thermo Fisher Scientific, Waltham, MA) and 100 units/ml of penicillin/streptomycin (EmbryoMax, Merck, Darmstadt, Germany) at 28^º^C with ambient CO_2_. The African green monkey kidney cell line Vero (ATCC: CCL-81) and the Rhesus monkey kidney cell line LLC-MK_2_ (ATCC: CCL-7) were cultured in Dulbecco’s modified Eagle medium (DMEM; Thermo Fisher Scientific, Waltham, MA) supplemented with 5% heat-inactivated FBS and 100 units/ml of penicillin/streptomycin at 37^º^C with 5% CO_2_, while the human lung carcinoma cell line A549 (ATCC: CCL-185) was cultured in DMEM supplemented with 10% heat-inactivated FBS and 100 units/ml of penicillin/streptomycin at 37^º^C with 5% CO_2_.

ZIKV Thai strain SV0010/15 isolated during a retrospective study of Thai fever cases [49], dengue virus serotype 2 (DENV 2; strain 16,681) and Japanese encephalitis virus (JEV; strain Beijing 1) were used in this study. ZIKV was provided by the Armed Forces Research Institution of Medical Sciences (AFRIMS) and the Development of Disease Control, Ministry of Public Health, Thailand. All viruses were propagated in C6/36 cells and harvested after cytopathic effects (CPE) appeared in the cells. Cell debris was removed by centrifugation and the virus stock was adjusted to have a final concentration of 20% FBS before the virus was stored at -80^º^C. Identity of the viruses was confirmed by DNA sequencing after RT-PCR amplification, and the virus titers were quantitated by plaque assay.

### Plaque assay

Vero (ZIKV) or LLC-MK_2_ (DENV and JEV) cells were cultured in 6-well cell culture plates at 37 ^º^C with 5% CO_2_ for 22–24 h to obtain approximately 90% confluency. Before infection, cell culture supernatant (control) or stock virus were 10-fold serially diluted with BA-1 virus diluent (1× medium 199/Earle’s balanced salts, 0.05 M Tris-HCl [pH 7.6], 1% bovine serum albumin fraction V, 0.075% NaHCO3, 100 U of penicillin-streptomycin per ml). Cells were infected with 200 µl of the diluted cell culture supernatant or diluted virus at 37 ^º^C for 2 h with gentle rocking every 10 min. After that, the infected cells were overlaid with 4 ml of overlay medium (1.2% methylcellulose (Merck KGaA, Darmstadt, Germany) in DMEM supplemented with 2% FBS for ZIKV, or 4 ml of 0.8% Seakem LE agarose (Cambrex Bio Science Walkersville, Inc., Walkersville MD) in 1X nutrient solution for DENV 2 and JEV). The infected cells were incubated for a further 7 days at 37 ^º^C with 5% CO_2_. On day 7, the overlay medium was removed and infected cells were then fixed with 1 ml of 3.7% formaldehyde (Merck KGaA, Darmstadt, Germany) in PBS for at least an hour at room temperature. After that, the formaldehyde was removed and the cells were washed with water prior to staining cells with 1% crystal violet (Merck KGaA, Darmstadt, Germany) in 20% ethanol. All samples were assayed in duplicate.

### Compounds

Baicalein (Supplemental Fig. 1; Merck KGaA, Darmstadt, Germany) was dissolved in 100% DMSO (Merck KGaA, Darmstadt, Germany) to give a stock solution concentration of 100 mM, which was stored at -30 ^º^C until used. Working solutions were prepared by diluting the stock solution in complete cell culture medium to the desired concentration. Orlistat (Merck KGaA, Darmstadt, Germany) was dissolved in 100% DMSO to give a stock concentration of 38.3 mM and was also stored at -30^o^C until used.

### MTT assay

A549 cells were cultured in 96-well cell culture plates at 37 ^º^C with 5% CO_2_ for 24 h prior to treating cells with 100 µl of serially increasing concentrations (1-800 µM) of baicalein or 1-400 µM orlistat. Positive control (20% ethanol), negative control (only complete media), and vehicle control (0.001-0.8% DMSO for baicalein and 0.003-1% for orlistat) were included, and all of the samples were evaluated in quadruplicate. Cells were incubated with the compound for 24 h under standard conditions. Then, 12.5 µl of 5 mg/ml MTT solution (Thiazolyl blue tetrazolium bromide; ITW Reagents, Barcelona, Spain) in PBS was added and cells which were incubated for a further hour under the same conditions. After that, cell culture supernatants were removed followed by replacement with 100 µl of 100% DMSO and incubation for another hour under the same conditions. The absorbance was measured at 570 nm using a Beckman Coulter DTX 880 multimode detector (Beckman Coulter, Brea, CA) using the Multimode analysis software version 3.3.0.9 (Beckman Coulter, Brea, CA). The half-maximal cytotoxic concentration (CC_50_) was calculated using the freeware ED50plus (v1.0) software (http://sciencegateway.org/protocols/cellbio/drug/data/ed50v10.xls).

### Trypan blue exclusion assay

A549 cells were cultured in 24-well cell culture plates under standard conditions for 24 h. Cells were treated with various concentrations (1-800 µM) of baicalein in duplicate. At 24 h after treatment, cells were visualized under an inverted microscope prior to harvesting cells by brief trypsinization with 0.25% trypsin/0.1% EDTA. Harvested cell suspensions were then mixed with 0.4% trypan blue solution (Merck KGaA, Darmstadt, Germany) in PBS. Live and dead cells were counted using a hemocytometer.

### Viral inactivation activity testing

Stock virus was directly incubated with baicalein at 37 ^º^C at final concentrations of 100, 250 or 500 µM for the desired times (1, 3, 6 h). Negative control (only DMEM) and vehicle control (0.1, 0.25, 0.5% DMSO) incubations were performed in parallel, and all experiments were undertaken independently in triplicate. After incubation, the virus mixtures were 10-fold serially diluted with BA-1 virus diluent prior to adding 200 µl of diluted viruses onto the cells in order to determine virus titer by plaque assay.

### Virus infection

A549 cells were cultured in 6-well cell culture plates at 37 ^º^C with 5% CO_2_ for 24 h. Cell culture supernatants were aspirated away prior to infecting cells with 1 ml of ZIKV with a multiplicity of infection (MOI) of 1 for 2 h under standard conditions. Virus was prepared in DMEM without FBS and penicillin/streptomycin supplementation. After that, the inoculum was removed and the cells were washed once with PBS. Subsequently, 2 ml of complete medium with or without compound as appropriate was added followed by incubation for a further 24 h under standard conditions.

### Determination of the half-maximal effective concentration (EC_50_)

A549 cells were cultured in 6-well cell culture plates for 24 h under standard conditions prior to being infected with ZIKV at MOI 1. At 2 h post-infection, the inoculum was removed and cells were washed once with PBS followed by treating cells with 2 ml of various concentrations (1-200 µM) of baicalein in culture medium. Cells were incubated for 24 h under standard conditions. At 24 h post-treatment, cell culture supernatants were harvested in order to determine the viral titer by plaque assay. Untreated control (only complete medium) and vehicle control (0.001-0.2% DMSO) were undertaken in parallel. The EC_50_ was calculated using the freeware ED50plus (v1.0) software (http://sciencegateway.org/protocols/cellbio/drug/data/ed50v10.xls).

### Time-of-addition analysis

A549 cells were cultured in triplicate in 6-well cell culture plates for 24 h under standard conditions. For pre-treatment studies, cells were pre-treated with 2 ml of 100 µM baicalein for 1, 3, or 6 h under standard conditions, after which cells were washed once with PBS and then infected with ZIKV at MOI 1. After infection, the virus was removed and cells were washed once with PBS, before incubating cells with 2 ml of complete medium for 24 h under standard conditions. For co-treatment studies, baicalein and ZIKV (or ZIKV and vehicle control) were prepared together in DMEM (without FBS or penicillin/streptomycin) in a total volume of 2 ml with a final concentration of 100 µM of baicalein and ZIKV at MOI 1. The mixture of virus and compound was added to the cells immediately after preparation. Cells were incubated for 2 h under standard conditions prior to removal of the mixture of virus and compound (or vehicle control). Cells were washed once with PBS before adding 2 ml of complete medium and incubating cells for a further 24 h under standard conditions. For post-treatment studies, cells were infected with MOI 1 of ZIKV for 2 h under standard conditions, after which the virus was removed, cells were washed once with PBS, and 2 ml of complete medium was added. After that, 2 ml of 100 µM of baicalein (or vehicle control) were used to replace the media at 0, 1, 3, 6, or 9 h after infection, and cells were incubated further under standard conditions until 24 h post infection. Mock-infected, untreated control (only complete medium), and vehicle control (0.1% DMSO) were undertaken in parallel and all experiments were undertaken as independent triplicates.

### Flow cytometry

Infected or mock infected cells were blocked with 100 µl of 10% goat serum (Thermo Fisher Scientific, Waltham, MA) in PBS for 30 min on ice and then washed once with 800 µl of 1% BSA in PBS. Cells were fixed with 200 µl of 4% paraformaldehyde (Merck KGaA, Darmstadt, Germany) in PBS in the dark for 20 min at room temperature followed by washing twice with 800 µl of 1% BSA in PBS. Fixed cells were permeabilized with 200 µl of 0.2% Triton X-100 (Merck KGaA, Darmstadt, Germany) in PBS in the dark for 10 min at room temperature. Then permeabilized cells were washed once with 800 µl of 1% BSA in PBS followed by incubating cells with a 1:2 dilution of mouse anti-flavivirus E protein antibody HB112 [50] in 1% BSA in PBS at 4 ^º^C for overnight. Cells were washed twice with 800 µl of 1% BSA in PBS. Cells were then incubated with a 1:40 dilution of fluorescein isothiocyanate (FITC)-conjugated goat anti-mouse IgG (5230 − 0307; SeraCare, Milford, MA) in 1% BSA in PBS at room temperature for an hour. Cells were washed twice with 800 µl of 1% BSA in PBS and were resuspended with PBS. Finally, fluorescent signal was analyzed by flow cytometry on a BD FACalibur™ Flow Cytometer System (Becton Dickinson, Franklin Lakes, NJ).

### Quantitative RT-PCR (qRT-PCR)

Total RNA was extracted from 500 µl of cell culture supernatant using 500 µl of TRIzol reagent (Ambion, Waltham, MA). Total RNA concentration was quantified by a Nanodrop 2000 spectrophotometer (Thermo Fisher Scientific, Waltham, MA). Then, 90 ng of the total RNA was used as a template for cDNA synthesis by RT-PCR. First strand of cDNA was synthesized using random hexamer primers (Thermo Fisher Scientific, Waltham, MA) and RevertAid Reverse Transcriptase (Thermo Fisher Scientific, Waltham, MA). Subsequently, cDNA was amplified by PCR using specific primers. Quantitative PCR (qPCR) was performed based on SYBR technique using KAPA SYBR® FAST qPCR Master Mix (2X) kit (Kapa Biosystems, Wilmington, MA). ZIKV genome was quantitated using specific primers under these conditions; 95 ^º^C for 3 min followed by 40 cycles of denaturation at 95 ^º^C for 10 s, annealing at 60 ^º^C for 30 s, and extension at 72 ^º^C for 20 s and followed by a dissociation curve analysis at 95 ^º^C for 15 s, 60 ^º^C for 15 s, and 95 ^º^C for 15 s with fluorescent detection in a Mastercycler® ep realplex real-time PCR system (Eppendorf AG, Hamburg, Germany).

- Forward primer for conventional PCR: 5’-AGG GAA TAC ACG AAC CGG AT-3’
- Reverse primer for conventional PCR: 5’-TAA GGC CAA GCA CAT AAG GGA-3’
- Forward primer for qPCR: 5’-TTG GAG GAA TGT CCT GGT TCT CAC-3’
- Reverse primer for qPCR: 5’-AGT CAG GAT GGT ACT TGT ACC-3’

### Western blotting

Total proteins were extracted from infected or mock infected cells using RIPA lysis buffer containing protease inhibitor cocktail (Merck KGaA, Darmstadt, Germany) and the total protein concentration was then measured by the Bradford protein assay. A total of 30 µg of total proteins were separated through 12% discontinuous SDS-PAGE gels followed by blotting onto nitrocellulose membranes (GE Healthcare Life Science, Chicago IL). The membranes were blocked with 5% skim milk (Fonterra, Selangor, Malaysia) in TBS-T at room temperature for 30 min before probing with an appropriate primary antibody prepared in 5% skim milk in TBS-T at 4 ^º^C for overnight. Primary antibodies used included a 1:500 dilution of a mouse anti-flavivirus E protein antibody (HB112), a 1:1,000 dilution of a rabbit anti-Zika NS1 antibody (GTX133307; GeneTex, Inc, Irvine, CA), and a 1:5,000 dilution of mouse anti-GAPDH antibody (sc-32,233; Santa Cruz Biotechnology, Dallas, TX). Membranes were then washed with TBS-T for 5 min for 3 times prior to probing with an appropriate secondary antibody at room temperature for an hour. Secondary antibodies used included a 1:5,000 dilution of a horseradish peroxidase (HRP)-conjugated goat anti-mouse IgG (A4416; Merck KGaA, Darmstadt, Germany), and a 1:8,000 dilution of a HRP-conjugated goat anti-rabbit IgG (31,460; Thermo Fisher Scientific, Waltham, MA). The signals were developed using a western blot substrate (Immobilon Forte Western HRP Substrate; Merck KGaA, Darmstadt, Germany) combined with an in-house 4IPBA-ECL solution [51] with the proportion of 1:3 for E protein and a proportion of 1:10 for NS1 and GAPDH proteins. The chemiluminescent signals were detected under an Azure c400 visible fluorescent western blot imaging system (Azure Biosystems Inc, Dublin, CA). Uncropped western blots can be found in the Supplemental materials file.

### Indirect immunofluorescence assay (IFA)

Cells on glass coverslips were washed with PBS prior to fixing with 4% paraformaldehyde in PBS at room temperature for 20 min. After that, cells were washed three times with PBS followed by permeabilization with 0.3% Triton X-100 in PBS at room temperature for 10 min followed by washing three times with 0.03% Triton X-100 in PBS. Cells were then blocked with 5% BSA in PBS for 30 min before incubating with a mixture of two primary antibodies in 0.03% Triton X-100 in PBS at 4 ^º^C for overnight. Primary antibodies were a 1:2 dilution of a mouse pan-specific anti-flavivirus E antibody (HB112) and a 1:100 dilution of a rabbit anti-Zika NS1 antibody (GTX133307; GeneTex, Inc, Irvine, CA). Subsequently, cells were washed 3 times with 0.03% Triton X-100 in PBS before incubating with a mixture of two secondary antibodies and 1:500 dilution of 4′,6-diamidino-2-phenylindole dihydrochloride (DAPI; Merck KGaA, Darmstadt, Germany) in 0.03% Triton X-100 in PBS at room temperature for an hour. Secondary antibodies were a 1:200 dilution of Alexa Flour® 488 conjugated donkey anti-mouse IgG (A21202; Thermo Fisher Scientific, Waltham, MA) and a 1:50 dilution of Rhodamine Red^TM^-X-conjugated goat anti-rabbit IgG (111-295-144; Jackson ImmunoResearch Inc, West Grove, PA). Cells on coverslips were washed three times with 0.03% Triton X-100 in PBS followed by mounting the coverslips on the glass slide with ProLong Gold antifade reagent (Thermo Fisher Scientific, Waltham, MA). The fluorescent signals were visualized under a LSM 800 confocal laser scanning microscope (Carl Zeiss Microscope GmbH, Jena, Germany) with a magnification of 100x equipped with the ZEN (blue edition) version 2.3 (Carl Zeiss Microscope GmbH, Jena, Germany) software.

### Statistical analysis

The data were analyzed and plotted using GraphPad Prism version 5.01 for Windows (GraphPad Software, La Jolla, CA) with error bars representing mean ± SD. Statistical significance analysis was undertaken by One-Way ANOVA with Tukey’s Multiple Comparison Test (GraphPad Software, La Jolla, CA) or by (for western blots) independent sample T-test in PASW statistics 18 (PASW Statistics for Windows, Version 18.0. SPSS Inc., Chicago, IL) with a *p*-value < 0.05 for significance.

## Results

### Cytotoxicity of baicalein

**Fig. 1:**
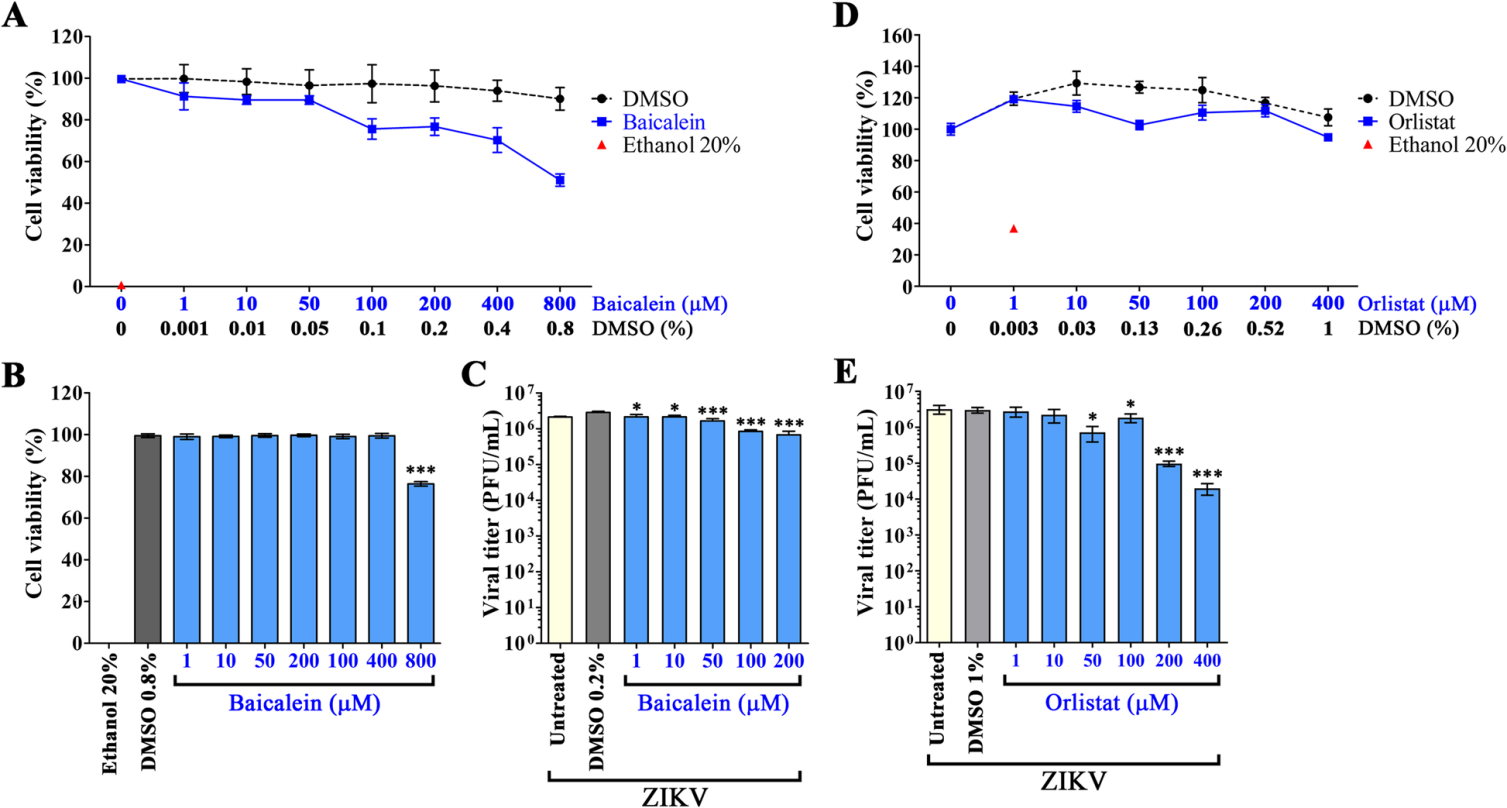
Cytotoxicity and EC_50_ of baicalein and orlistat towards A549 cells. **A**, **B** A549 cells were treated with varying concentrations (1-800 µM) of baicalein for 24 h. Cell viability was determined by (**A**) the MTT assay and (**B**) a trypan blue exclusion assay. Experiments were undertaken independently in quadruplicate. **C** A549 cells were infected with ZIKV with MOI 1 and treated with varying concentrations (1-200 µM) of baicalein for 24 h. Viral titer was determined from cell culture supernatant by plaque assay to calculate the EC_50_. **D**, **E** A549 cells were treated with varying concentrations (1-400 µM) of orlistat for 24 h. Cell viability was determined by (**D**) the MTT assay. Experiments were undertaken independently in quadruplicate. **E** A549 cells were infected with ZIKV with MOI 1 and treated with varying concentrations (1-400 µM) of orlistat for 24 h. Viral titer was determined from cell culture supernatant by plaque assay to calculate the EC_50_. Experiments were undertaken in triplicate with duplicate plaque assay. Error bars show mean ± SEM and *p*-value compared to DMSO control (* *p*-value < 0.05, ** *p*-value < 0.01, *** *p*-value < 0.001)

To determine the cytotoxicity of baicalein, A549 cells were incubated with 1, 10, 50, 100, 200, 400, and 800 µM of baicalein for 24 h, prior to determining cell viability. Results showed that the cell viability of baicalein-treated cells as assessed by the MTT assay decreased in a dose-dependent manner (Fig. 1A). Determination of the half-maximal cytotoxic concentration showed that the CC_50_ was > 800 µM. Cell viability as assessed by a trypan blue exclusion assay showed approximately 100% cell viability at concentrations of baicalein between 1 and 400 µM, but viability was reduced by some 25% at a concentration of 800 µM (Fig. 1B).

### Evaluation of the EC_50_ of baicalein

To determine the effect of baicalein on ZIKV infection, A549 cells were infected with ZIKV and then treated with baicalein over a range of 1-200 µM prior to the determination of viral titer by plaque assay at 24 h.p.i. Results showed that viral production decreased in a dose-dependent manner (Fig. 1C), with an EC_50_ of 124.88 µM.

### Reference compound (orlistat) analysis

**Fig. 2:**
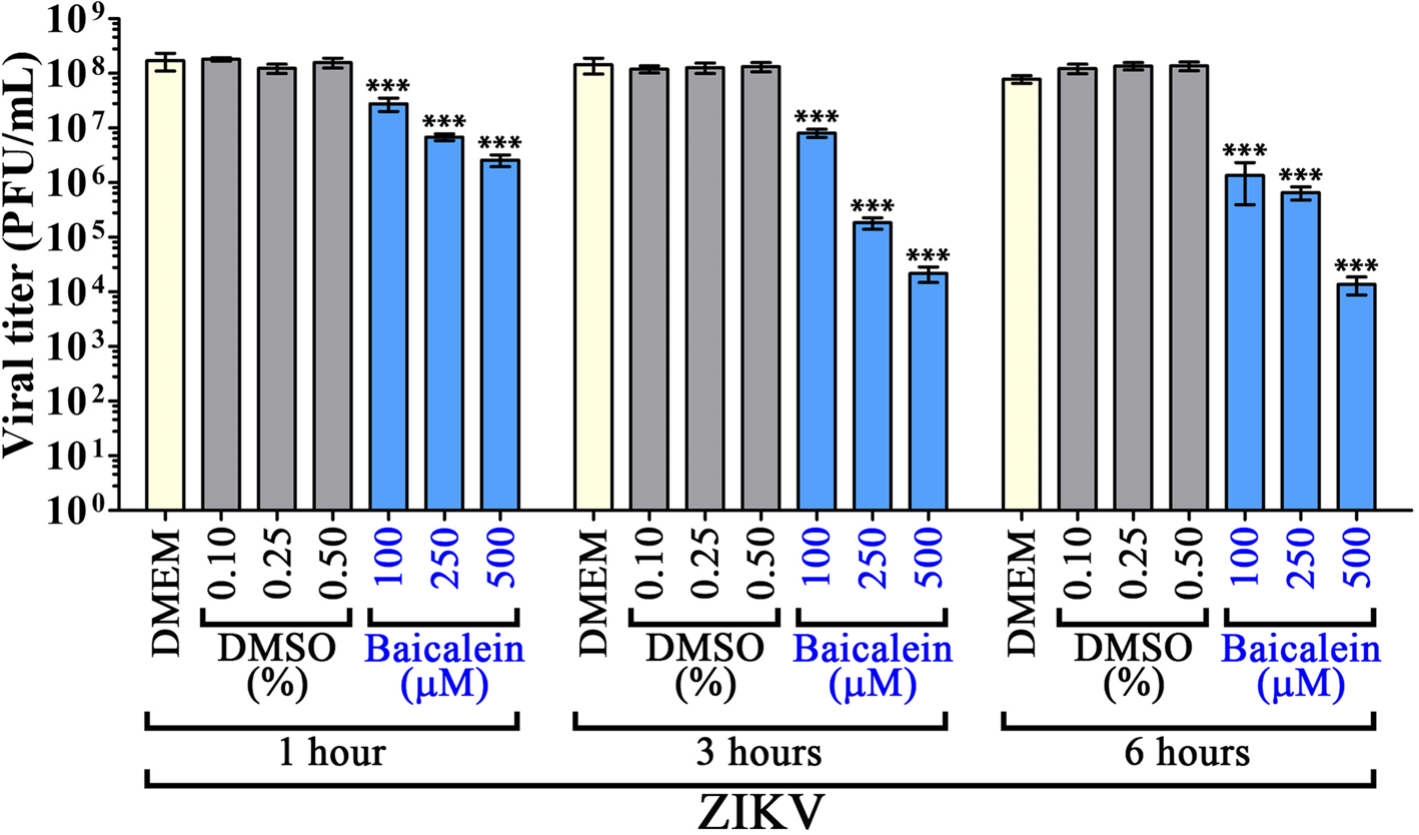
Virus inactivation activity of baicalein. Stock ZIKV was directly incubated with baicalein at final concentrations of 100, 250, and 500 µM or with an equivalent percentage of vehicle control at 37 ^º^C for 1, 3, or 6 h before titer was determined by plaque assay. Experiments were performed independently in triplicate with duplicate plaque assay. Error bars show mean ± SD and *p*-value compared to the appropriate DMSO control (* *p*-value < 0.05, ** *p*-value < 0.01, *** *p*-value < 0.001)

While there is no approved antiviral drug for ZIKV, we have previously shown anti-ZIKV activity of the FDA approved anti-obesity drug orlistat (tetrahydrolipstatin) [52], as well as established the EC_50_ of this drug against the closely related flavivirus, dengue virus (DENV) [53]. To provide a reference compound (positive control) analysis, we determined the cytotoxicity and anti-ZIKV activity of orlistat in A549 cells. A549 cells were incubated with 1, 10, 50, 100, 200 or 400 µM of orlistat for 24 h, prior to determining cell viability. Results showed that the cell viability of orlistat-treated cells as assessed by the MTT assay decreased in a dose-dependent manner (Fig. 2A). Determination of the half-maximal cytotoxic concentration showed that the CC_50_ was 1515.57 µM. To confirm the effect of orlistat on ZIKV infection, A549 cells were infected with ZIKV and then treated with orlistat over a range of 1-400 µM prior to the determination of viral titer by plaque assay at 24 h.p.i. Results showed that viral production decreased in a dose-dependent manner (Fig. 2B), with an EC_50_ of 109.68 µM, giving a selectivity index (SI) of 13.82. These results were consistent with our previous observations [52, 53], and in particular the EC_50_ value for ZIKV is very close to that previously determined for DENV (84.79 µM at 24 h post-treatment; [53]).

## Viral inactivation activity of baicalein

**Fig. 3:**
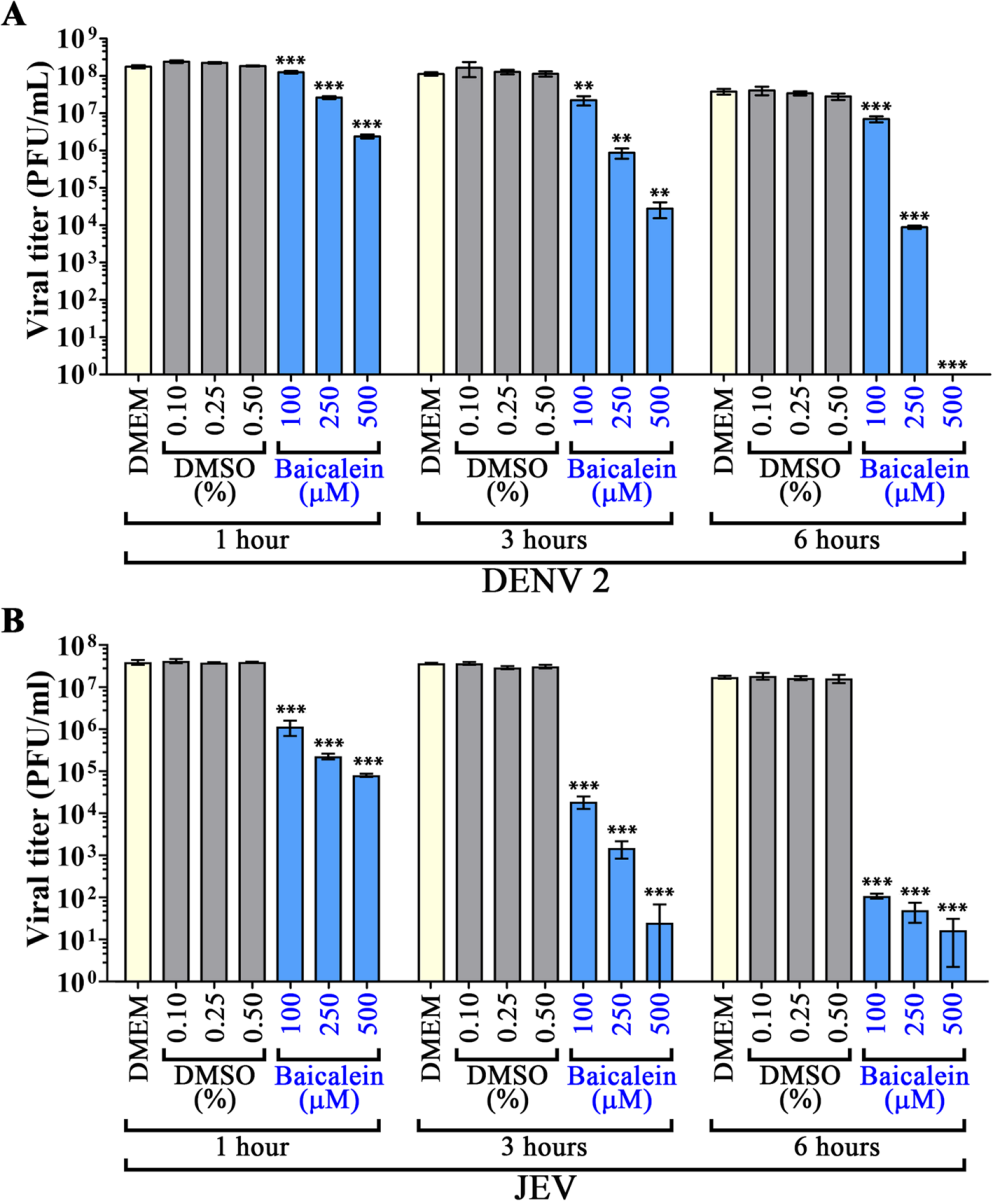
Virus inactivation activity of baicalein. **A** DENV 2, and **B** JEV were directly incubated with baicalein at final concentrations of 100, 250, and 500 µM or with an equivalent percentage of vehicle control at 37 ^º^C for 1, 3, or 6 h before titer was determined by plaque assay. Experiments were performed independently in triplicate with duplicate plaque assay. Error bars show mean ± SD and *p*-value compared to the appropriate DMSO control (* *p*-value < 0.05, ** *p*-value < 0.01, *** *p*-value < 0.001)

To determine any possible direct effect of baicalein on ZIKV virions, stock virus was directly incubated with baicalein at final concentrations of 100, 250, and 500 µM for 1, 3, and 6 h. The results showed that ZIKV titer decreased in a dose- and time-dependent manner (Fig. 2), although there were only modest further reductions in titer when the incubation time was increased from 3 to 6 h. However, at the highest incubation concentration (500 µM) and longest incubation time (6 h), virus titer was reduced by more than 4Log_10_ (Fig. 2). Given the significant viral inactivation activity of baicalein towards ZIKV, the experiment was repeated with both DENV 2 and JEV. The results (Fig. 3A, B) again showed direct inhibitory effects, with the effects being greater than had been observed for ZIKV. After 6 h incubation at the highest concentration of baicalein (500µM) no infectious virions were detected for DENV 2, while the JEV titer was reduced by some 7Log_10_. While the most dramatic effects on virus titers were seen with the highest concentration and longest incubation time, significant reductions in virus titers were seen for all three viruses with the shortest incubation time (1 h) and lowest concentration (100µM).

## Effects of baicalein on ZIKV infection

In order to further explore the anti-ZIKV activity of baicalein, three different treatment options (namely pre-treatment, co-treatment, and post-treatment) were investigated. In all cases a standard treatment of 100 µM baicalein or vehicle control (0.1% DMSO) was used, and all samples were collected at 24 h.p.i. Subsequently, viral infectivity, viral production, viral genome copy number, and viral protein expression were evaluated.

**Fig. 4:**
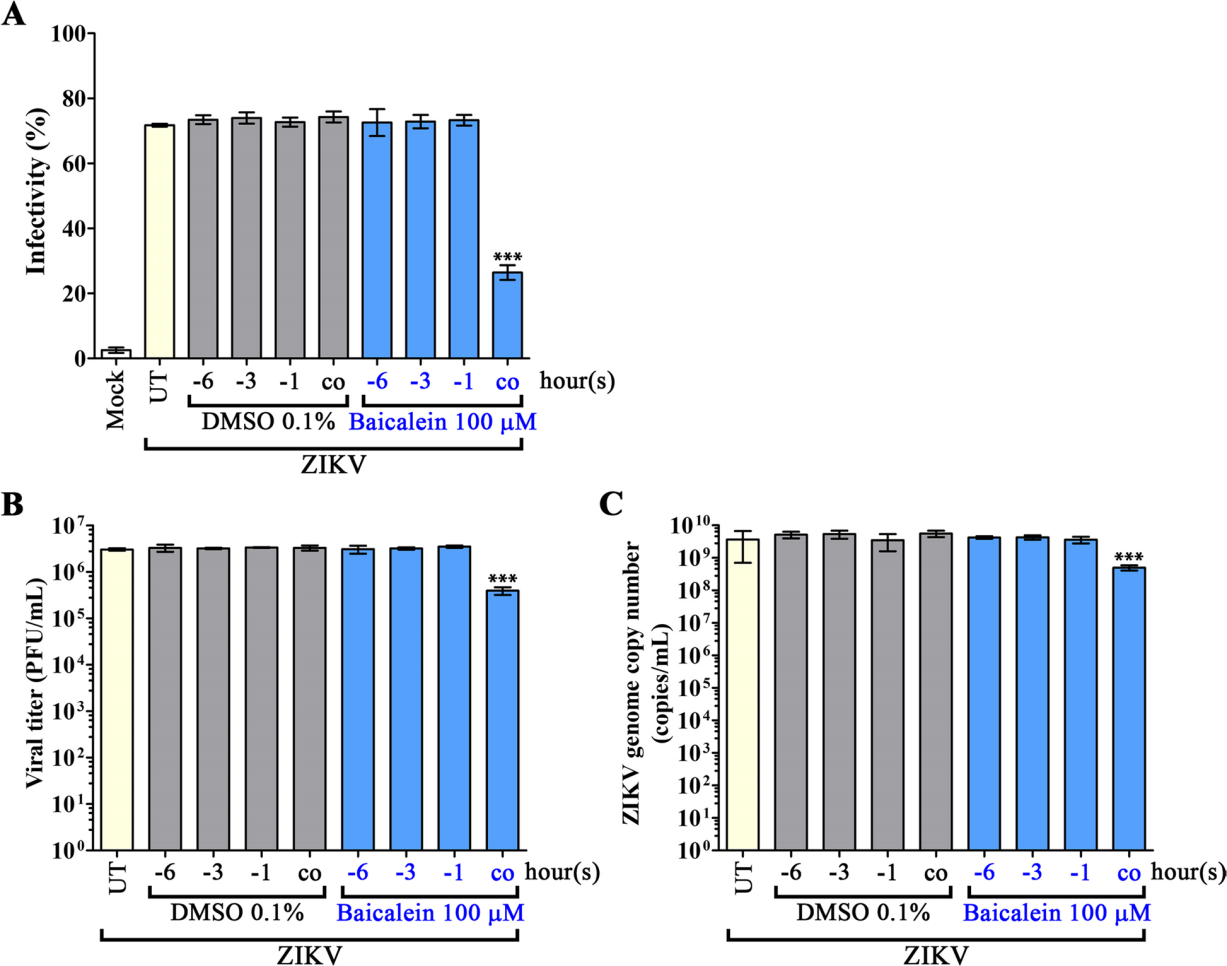
Effects of baicalein on the entry and adsorption stages of ZIKV infection. A549 cells were pre-treated with 100 µM baicalein for 1, 3, or 6 h (-1, -3, -6) before infection, or co-treated during infection. Cells were infected with MOI 1 of ZIKV and then infected cells were incubated for 24 h. Viral infectivity was determined by **A** flow cytometry, while **B** viral productivity was determined by plaque assay, and **C** viral genome copy number was determined by qRT-PCR. Viral titer and viral genome copy number are shown plotted on a logarithmic scale. Error bars show mean ± SD and *p*-value compared to DMSO control of each time-point (*** *p*-value < 0.001). UT = untreated

**Fig. 5:**
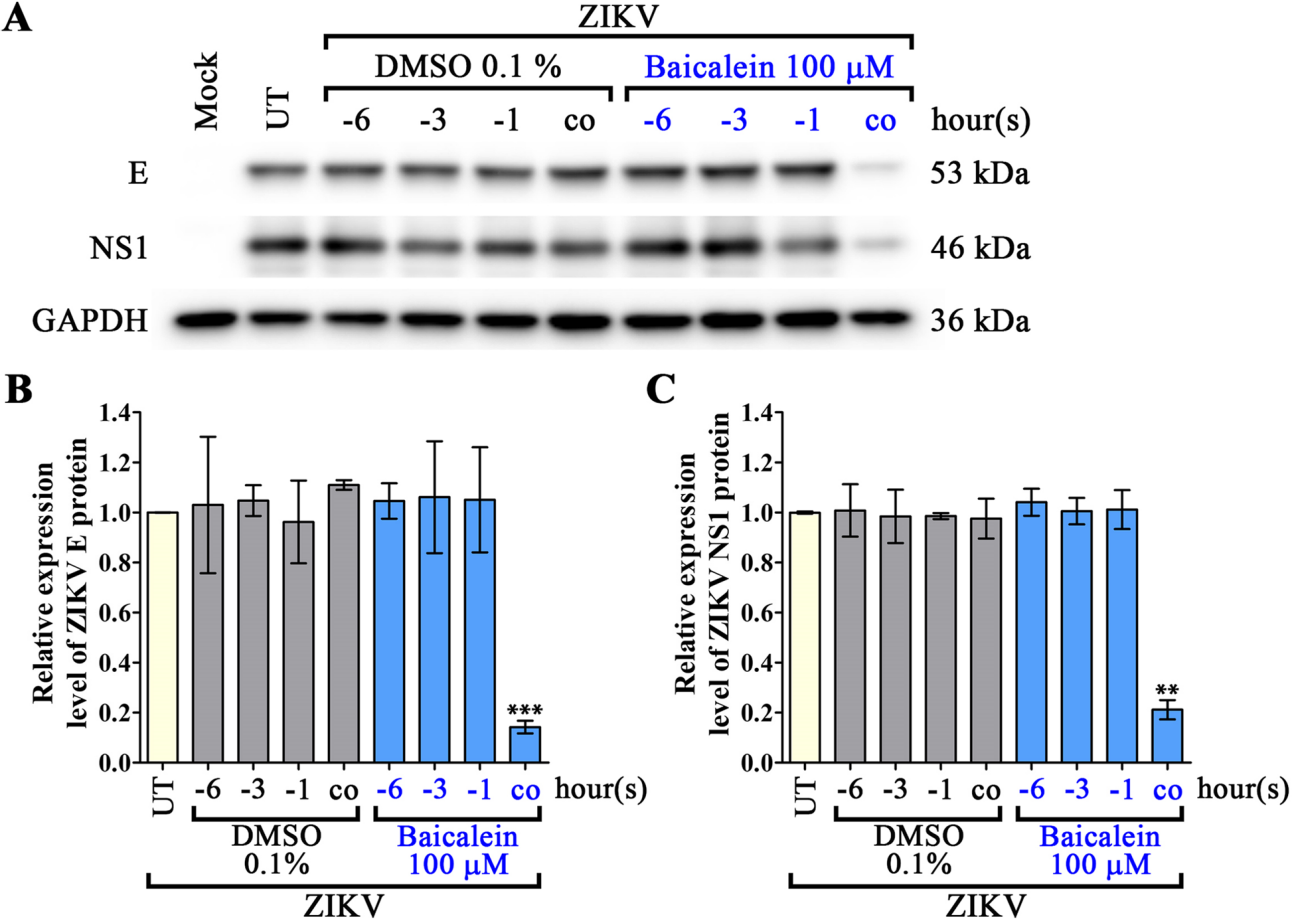
Effects of baicalein on viral protein expression at the entry and adsorption stage of ZIKV infection as assessed by western blotting. A549 cells were pre-treated with 100 µM baicalein at 1, 3, 6 h (-1, -3, -6) prior to infection, or cells were co-treated with baicalein during infection. Cells were infected with ZIKV at MOI of 1 prior to incubating cells for a further 24 h. Total proteins were extracted from cells and viral protein expression was examined by western blotting. **A** Viral structural (E) protein, viral non-structural (NS1) protein, and control GAPDH proteins were analyzed. **B**, **C** Intensity of the protein bands was quantitated and normalized against control GAPDH by ImageJ version 1.52a. Expression levels are shown as fold-change over the untreated sample. All experiments were undertaken independently in triplicate. Error bars show mean ± SD and *p*-value compared to the appropriate DMSO control of each time-point (** *p*-value < 0.01, *** *p*-value < 0.001). UT = untreated. Full uncropped blots of all replicates are available in the associated Supplementary materials

Any prophylactic activity of baicalein was determined by pre-treatment, in which A549 cells were treated with 100 µM of baicalein for 1, 3, and 6 h (-1, -3, -6) prior to the infection step. The results showed that pre-treatment of cells with baicalein prior to infection had no effect (as compared to vehicle control treated cells) on the level of infection (Fig. 4A), the amount of virus produced (Fig. 4B), viral genome copy number in the supernatant (Fig. 4C), or on viral protein expression as assessed by either western blot (Fig. 5) or IFA (Supplemental Fig. S2).

To evaluate any possible effects of baicalein at the step of viral adsorption/entry, A549 cells were infected in the presence of the drug, after which cells were cultured for 24 h without the drug being present in the culture medium. The results showed a significant reduction (as compared to vehicle control treated cells) in the percentage of infection (Fig. 4A) infectious particle production (Fig. 4B), and viral genome copy number (Fig. 4C). Viral protein expression as assessed by western blot was strongly reduced (approximately 80% for both E and NS1 proteins) as compared to control, vehicle treated cells (Fig. 5). Similarly, expression of both proteins was markedly reduced compared to control cells as assessed by IFA (Supplemental Fig. S2).

**Fig. 6:**
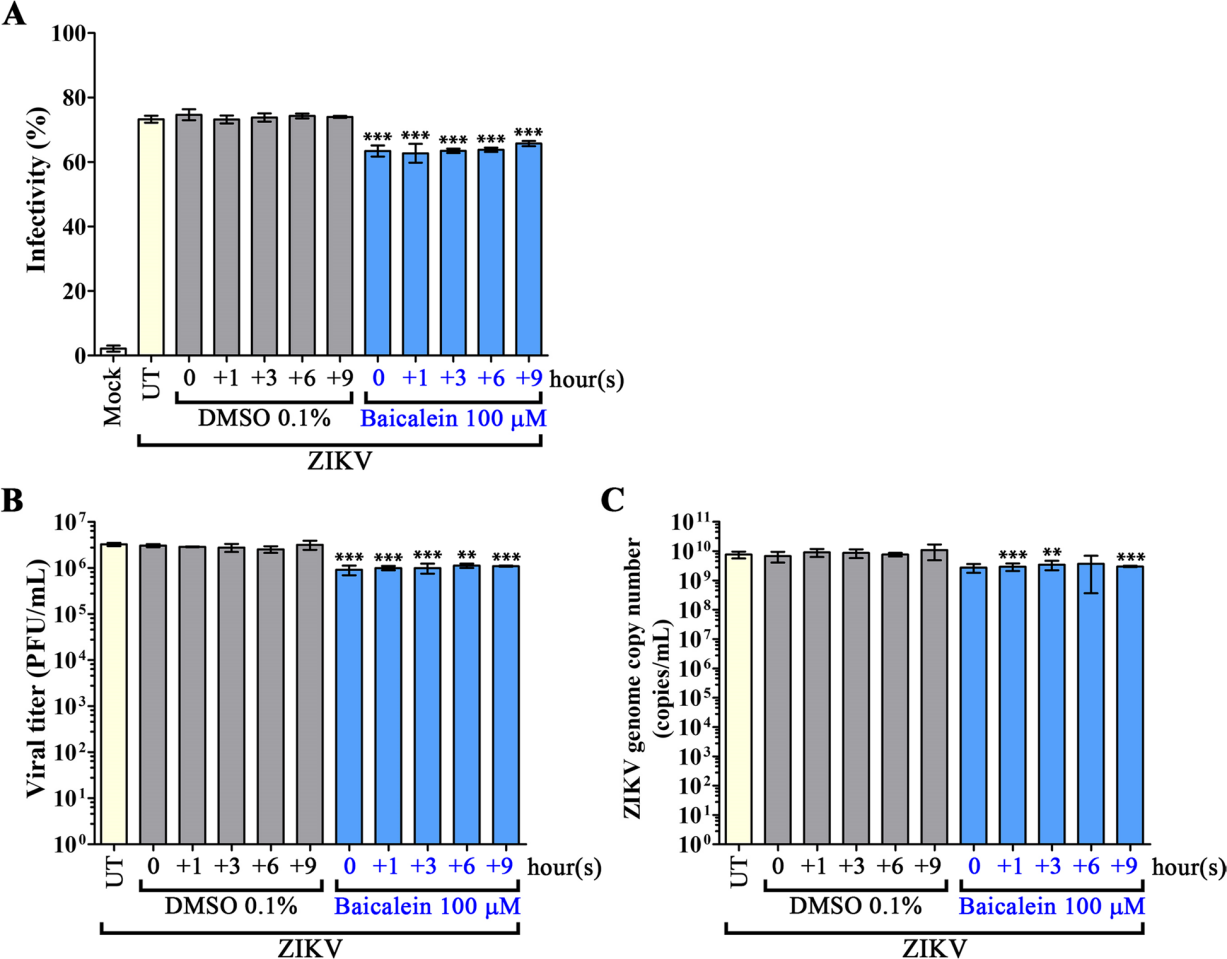
Effects of baicalein on post-adsorption stage of ZIKV infection. A549 cells were infected with MOI 1 of ZIKV prior to treating infected cells with 100 µM baicalein at 0, 1, 3, 6, 9 h (0, + 1, +3, + 6, +9) after infection. Cells were incubated until 24 h post infection. Viral infectivity was determined by flow cytometry (**A**), viral productivity was determined by plaque assay (**B**), and viral genome copy number was determined by qRT-PCR (**C**). Viral titer and viral genome copy number are shown in a logarithmic scale. Error bars show mean ± SD and *p*-value compared to DMSO control of each time-point (* *p*-value < 0.05, ** *p*-value < 0.01, *** *p*-value < 0.001). UT = untreated

**Fig. 7:**
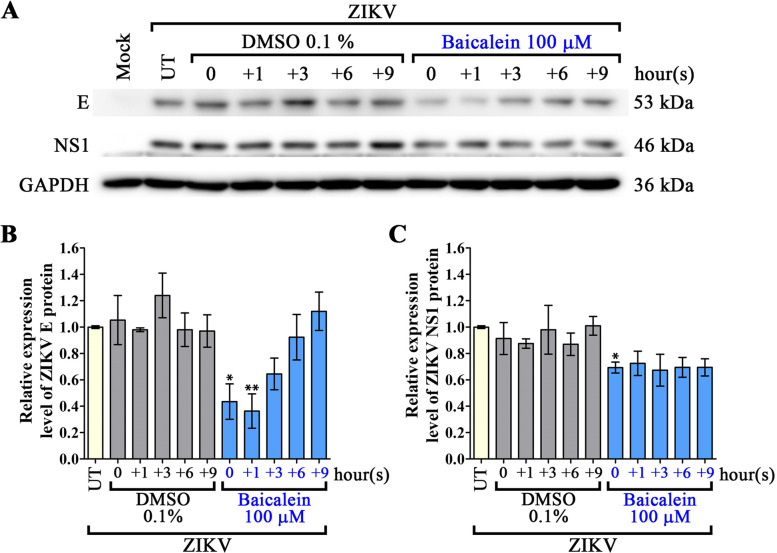
Effects of baicalein on viral protein expression at the post-adsorption stage of ZIKV infection assessed by western blotting. A549 cells were infected with MOI 1 of ZIKV prior to treating infected cells with 100 µM baicalein at 0, 1, 3, 6, 9 h (0, + 1, +3, + 6, +9) after infection. Cells were incubated further until 24 h post infection. Total proteins were extracted from cells and viral protein expression was examined by western blotting. Viral structural (E) protein, viral non-structural (NS1) protein, and control GAPDH protein were analyzed. Intensity of the protein bands was quantitated and normalized with control GAPDH by the ImageJ version 1.52a. The expression levels are shown in fold-change over the untreated sample (**B**, **C**). All experiments were undertaken in triplicate. Error bars show mean ± SD and *p*-value compared to DMSO control of each time-point (* *p*-value < 0.05, ** *p*-value < 0.01). UT = untreated. Full uncropped blots of all replicates are available in the associated Supplementary materials

Post-infection activity of baicalein was investigated by treating ZIKV-infected A549 cells with 100 µM of baicalein at 0, 1, 3, 6, and 9 h (0, + 1, +3, + 6, +9) post-infection, in parallel with vehicle control treated cells. The results showed a significant reduction under all drug treatments (as compared to vehicle control treated cells) with respect to level of virus infection (Fig. 6A), infectious particle production (Fig. 6B) and genome copy number (Fig. 6C). Viral E and NS1 protein expression assessed by western blot analysis showed that both proteins were significantly deceased at the 0 h time point, but that only E protein was significantly reduced at the + 1 h treatment time point (Fig. 7). No statistical difference in protein expression levels was observed at later treatment time points (Fig. 7). Viral protein expression as assessed by IFA showed slight reductions in E protein or NS1 protein compared to the DMSO control for each time-point (Supplemental Fig. S3), but this was not formally quantitated.

## Discussion

Among the more than 70 flaviviruses, ZIKV is an important virus that can cause serious autoimmune complications after infection, as well as congenital Zika syndrome in developing fetuses. While several vaccines are in the developmental pipeline, and a number of possible antiviral agents have been evaluated, there is still a lack of a commercially available vaccine, or specific therapeutic treatment (reviewed in [54]).

Baicalein, an aglycone of baicalin, is a flavone that has been proposed to have a number of properties including antioxidant, anticancer, anti-inflammation, antibacterial, and antiviral activities. Baicalein has been reported to be well absorbed in the rat gastrointestinal system [55], and to be rapidly absorbed in human subjects [56, 57]. However, it is also rapidly excreted with up to nearly 30% being found unchanged in the feces of human subjects [56, 57]. In addition, pharmacokinetic studies of baicalein in human subjects have shown low adverse events or clinical signs, even at a high dose [56, 57], which collectively have suggested that baicalein is safe and tolerable for human use.

Prior studies have shown baicalein to have activity against a number of mosquito transmitted viruses. A previous study showed an inhibitory effect towards DENV in African green monkey kidney Vero cells at the adsorption and post-adsorption stage with IC_50_ values of 7.14 µg/ml (~ 26.42 µM) and 6.46 µg/ml (~ 23.9 µM), respectively [46], with continuous treatment of baicalein from 5 h prior to infection to 4 days post infection showing an inhibitory effect towards DENV with an IC_50_ of 5.39 µg/ml (~ 19.95 µM) [46]. In addition, baicalein has shown antiviral activity against JEV in Vero cells at the entry, adsorption, and post-adsorption stage with IC_50_ values of 84.18 µg/ml (~ 311.5 µM), 7.27 µg/ml (~ 26.79 µM), and 14.28 µg/ml (~ 52.84 µM), respectively [45]. Baicalein also inhibited ZIKV in Vero cells at the entry, adsorption, and post-adsorption stage with IC_50_ values of 12, 20, and 0.004 µM, respectively [47]. Moreover, baicalein has also shown an antiviral effect against CHIKV at the entry, adsorption, and post-adsorption stages with the strongest effect being seen early in the post-adsorption stage [58].

In this study, we showed the anti-ZIKV activity of baicalein in human lung carcinoma A549 cells. While lung cells do not represent a target of ZIKV infection, these cells are commonly used in studies on ZIKV as they are both susceptible and permissive to ZIKV, and as such represent a valuable model system. The results from this study showed that baicalein inhibited ZIKV infection in A549 cells at the adsorption and post-adsorption stages. However, no prophylactic activity against ZIKV was observed, which contradicts previous studies. This discrepancy may arise from a number of factors including the type of cell line used in the investigation. Previous studies on DENV [46], JEV [45], CHIV [58] and ZIKV [47] used the non-human cell line Vero, while this study used the human cell line A549, and it has been proposed that differences in cell lines might affect the reported antiviral response induced by RNA viruses [59]. This is consistent with our recent study that failed to detect an antiviral effect for kaempferol in human HEK293T/17 cells, but noted both pro- (for DENV) and anti- (for JEV) viral effects in hamster BHK-21 cells [48]. Other factors that could affect the reported antiviral activity of a compound include length of incubation, assay time points and method of quantitation.

This study also showed a direct virus inactivation activity of baicalein on ZIKV, JEV and DENV, consistent with the previous reports of virus inactivation activity against DENV and JEV [45, 46]. Interestingly, baicalin, a metabolite of baicalein, has also been shown to have direct virus inactivation activity to DENV [60], and ZIKV [61]. A previous study has reported that natural compounds including flavones could interact with the E protein of ZIKV by forming polar bonds between the oxygen atom in the hydroxyl group of the compound and the carboxyl groups of Glu, Arg, and Asp within the active site and fusion loop of ZIKV E protein [62], thus the apparent virus inactivation activity of baicalein, a flavone with trihydroxy groups, may result from an inhibition of viral fusion with the endocytic membrane that occurs during viral entry [63].

Even through baicalein exhibits antiviral activity towards several viruses, the mechanism by which the compound inhibits viral replication is still unknown. However, studies have reported that baicalein could bind to the HIV integrase [64] and reverse transcriptase [65] which are required for viral replication. In particular, it was reported that the trihydroxy groups of baicalein showed greater activity towards the viral reverse transcriptase that to the host DNA and RNA polymerases [65], suggesting that baicalein may interact with a ZIKV enzyme such as the RNA-dependent RNA polymerase (NS5). This is potentially seen in the post-treatment reduction in ZIKV genome copy number seen here.

Whether baicalein has true antiviral activity (in addition to its strong inhibitory activity) remains unclear. The cellular effects seen could possibly reflect the presence of baicalein in the supernatant, which would serve to reduce the ability of newly produced virions to infect other cells. However, previous studies in A549 cells (the cell line used in this study) have shown that baicalein affects a number of cellular pathways including PTEN/PI3K/NF-kB [66, 67] and DEPP/Gadd45a and MAPKs [68], and both of these pathways have been shown to modulate ZIKV replication [69, 70].

## Conclusions

Baicalein has now been shown to possess anti-ZIKV activity in a human cell line.

## Supplementary information

**Additional file 1.**

## Data Availability

All data generated or analysed during this study are included in this published article and its supplementary information files with the exception of raw reads (plaque assay and CC_50_) which are available from the corresponding author on reasonable request.

## Abbreviations

AFRIMS: Armed Forces Research Institution of Medical Sciences
ATCC: American Type Culture Collection
BHK: Baby hamster kidney
BSA: Bovine serum albumin
CC_50_: half-maximal cytotoxic concentration
CHIKV: Chikungunya virus
CPE: cytopathic effects
DAPI: 4′,6-diamidino-2-phenylindole dihydrochloride
DMSO: dimethyl sulfoxide
E: Envelope protein
EC_50_: half-maximal effective concentration
FBS: fetal bovine serum
HEK: Human embryonic kidney
JEV: Japanese encephalitis virus
MTT: (3-(4,5-dimethylthiazol-2-yl)-2,5-diphenyltetrazolium bromide)
NS: non-structural (protein)
ORF: open reading frame
PBS: Phosphate-buffered saline
PCR: polymerase chain reaction
RIPA: Radioimmunoprecipitation assay (buffer)
RT-PCR: reverse transcriptase polymerase chain reaction
SDS-PAGE: Sodium dodecyl-sulfate polyacrylamide gel electrophoresis
SI: selectivity index
WNV: West Nile virus
YFV: Yellow fever virus
ZIKV: Zika virus
