# Supplementary material for: Investigation of the activity of baicalein towards Zika virus
Source: BMC Complement Med Ther. 2023 May 3;23:143. doi: 10.1186/s12906-023-03971-4 (PMC10158012; doi:10.1186/s12906-023-03971-4)
Supplement: Supplementary file 1 — Additional file 1. [file 12906_2023_3971_MOESM1_ESM.pdf]

Investigation of the antiviral activity of baicalein towards Zika virus

Suteema Sawadpongpan[1], Janejira Jaratsittisin[1], Atitaya Hitakarun[1], Sittiruk Roytrakul[2], Nitwara Wikan[3] and Duncan R. Smith[1]*

[1]Institute of Molecular Biosciences, Mahidol University, Salaya, 73170, Thailand;

[2]National Center for Genetic Engineering and Biotechnology (BIOTEC), National Science and Technology Development Agency, Pathum Thani, 12120, Thailand

[3]Department of Pharmacology, Faculty of Medicine, Chiang Mai University, Chiang Mai, 50200, Thailand;

* Correspondence: duncan_r_smith@hotmail.com; Tel.: +66-2800-3624-8

ORCID

Janejira Jaratsittisin 0000-0002-0651-5377

Atitaya Hitakarun 0000-0002-3614-2313

Sittiruk Roytrakul 0000-0003-3696-8390

Nitwara Wikan 0000-0002-0708-0328

Duncan R. Smith 0000-0002-6592-9852

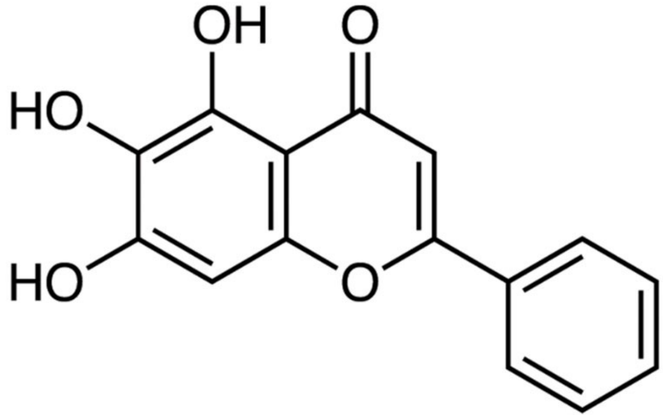

Supplemental Figure 1. The chemical structure of baicalein (5,6,7-trihydroxyflavone).

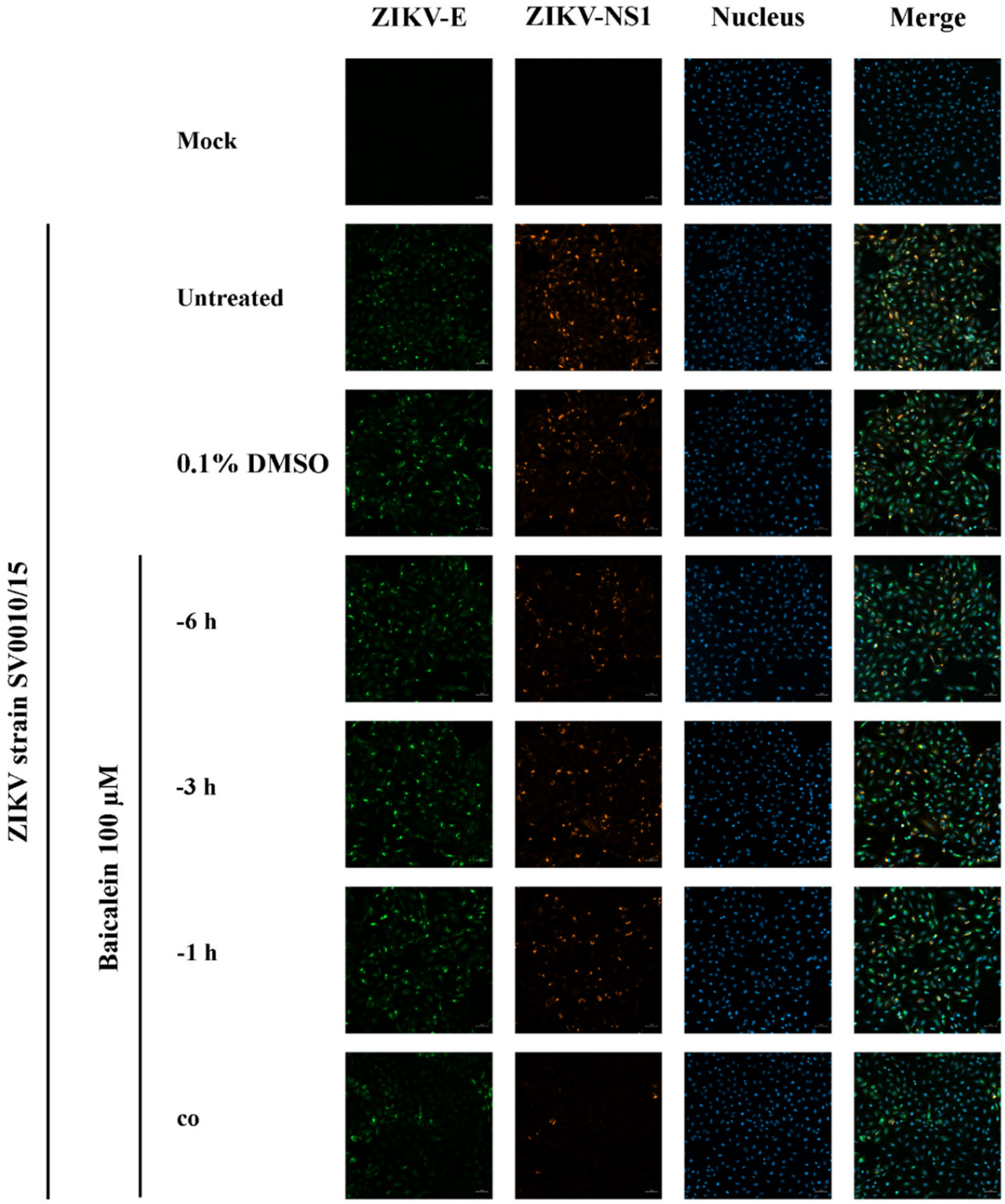

Supplemental Figure 2. Effects of baicalein on viral protein expression at the entry and adsorption stages of ZIKV infection assessed by IFA. A549 cells grown on glass coverslips were pre-treated with 100 μM baicalein for 1, 3, or 6 h (-1, -3, -6) prior to infection, or cells were co-treated with baicalein during infection. Cells were then infected with MOI 1 of ZIKV. At 24 h post infection, cells were stained with specific antibodies conjugated with fluorescence and DAPI for nuclear staining. Appropriate mock, untreated and DMSO treated controls were undertaken in parallel. Fluorescent signals were detected and visualized under a ZEISS LSM 800 with 100X magnification. Cellular nucleus (blue), viral E protein (green), and viral NS1 protein (red) were shown with non-contrast adjusted un-merged and merged images.

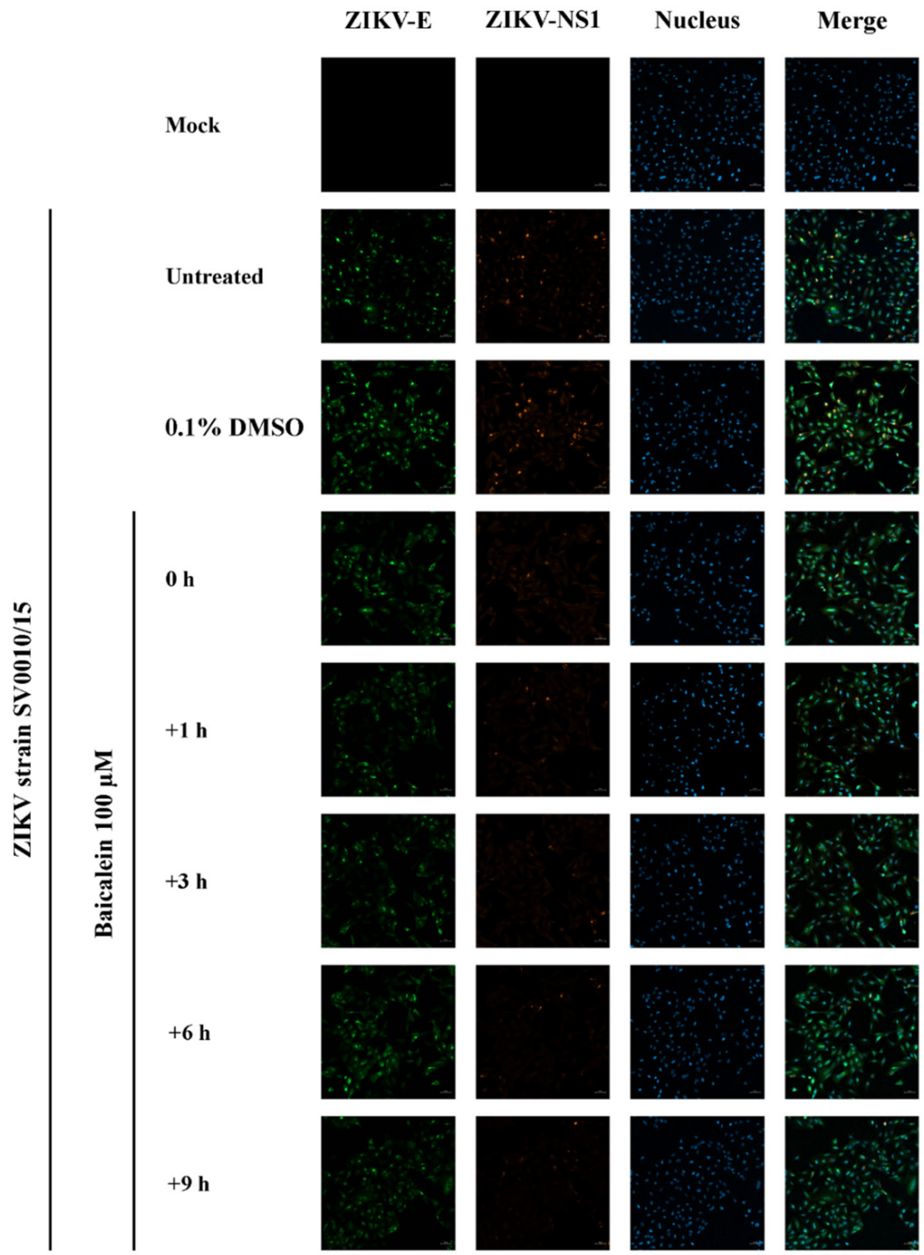

Supplemental Figure 3. Effects of baicalein on viral protein expression at the post-adsorption stage of ZIKV infection assessed by IFA. A549 cells grown on glass coverslips were infected with MOI 1 of ZIKV prior to treating infected cells with 100 µM baicalein at 0, 1, 3, 6, 9 hours (0, +1, +3, +6, +9) after infection. At 24 h post infection, cells were stained with specific antibodies conjugated with fluorescence and DAPI for nuclear staining. Appropriate mock, untreated and DMSO treated controls were undertaken in parallel. Fluorescent signals were detected and visualized under a ZEISS LSM 800 with 100X magnification. Cellular nucleus (blue), viral E protein (green), and viral NS1 protein (red) were shown with non-contrast adjusted un-merged and merged images.

Uncropped western blots

Western blot of viral protein expression at the entry and adsorption stage

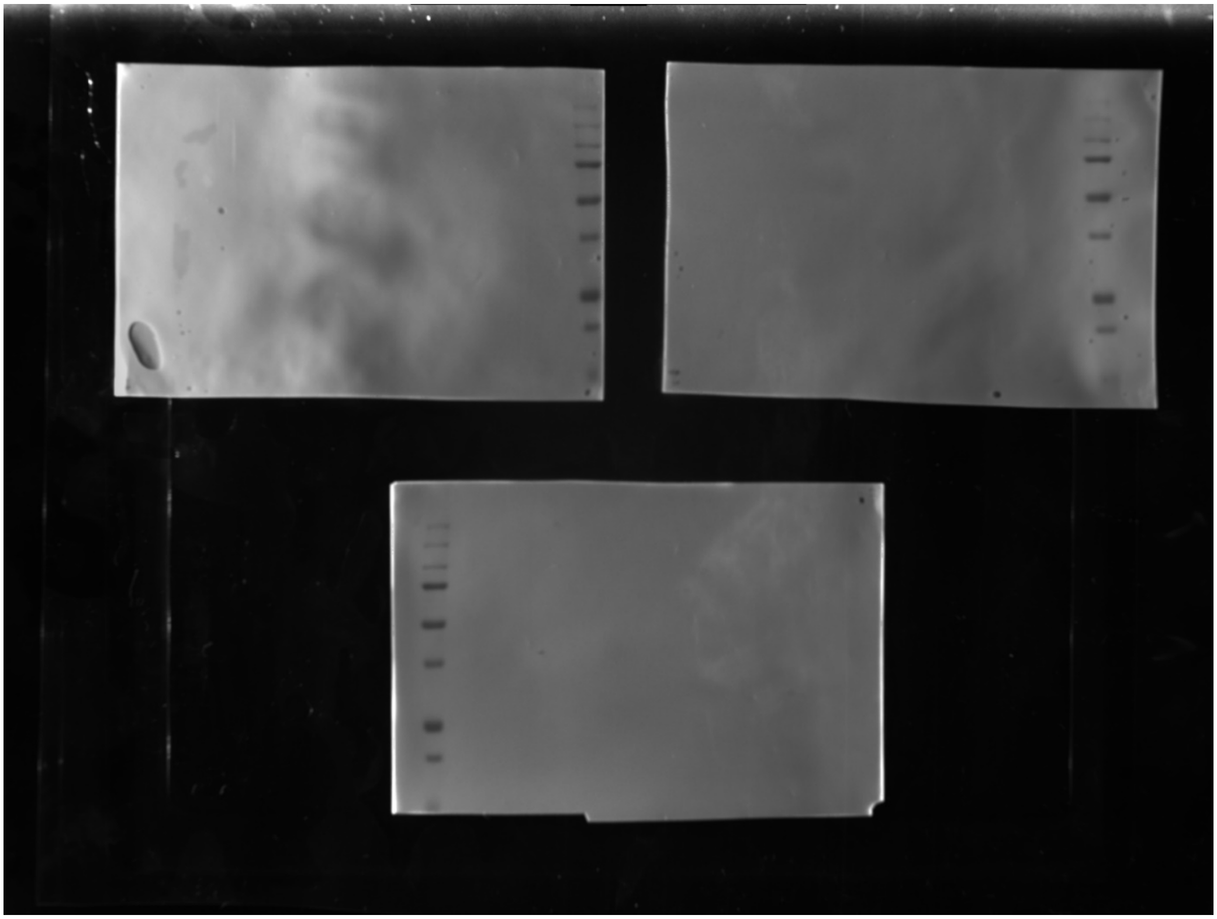

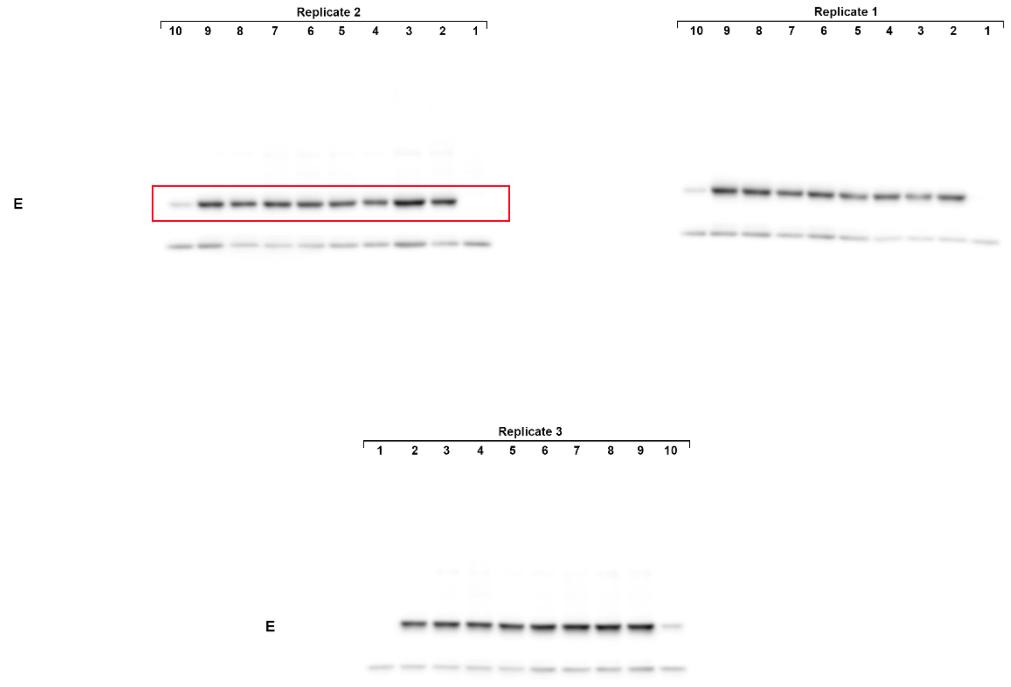

Full western blot membranes of E protein captured by Azure400 (Replicate 1-3), red boxes indicate bands presented in Fig 6; E protein. Replicate 1-2 were flipped horizontally for publication.

1: Mock
2: Untreated cells with ZIKV infection
3: Treated cells with 0.1% DMSO before ZIKV infection for 6 hours
4: Treated cells with 0.1% DMSO before ZIKV infection for 3 hours
5: Treated cells with 0.1% DMSO before ZIKV infection for 1 hour
6: Treated cells with 0.1% DMSO during ZIKV infection
7: Treated cells with 100 µl of Baicalein before ZIKV infection for 6 hours
8: Treated cells with 100 µl of Baicalein before ZIKV infection for 3 hours
9: Treated cells with 100 µl of Baicalein before ZIKV infection for 1 hour
10: Treated cells with 100 µl of Baicalein during ZIKV infection

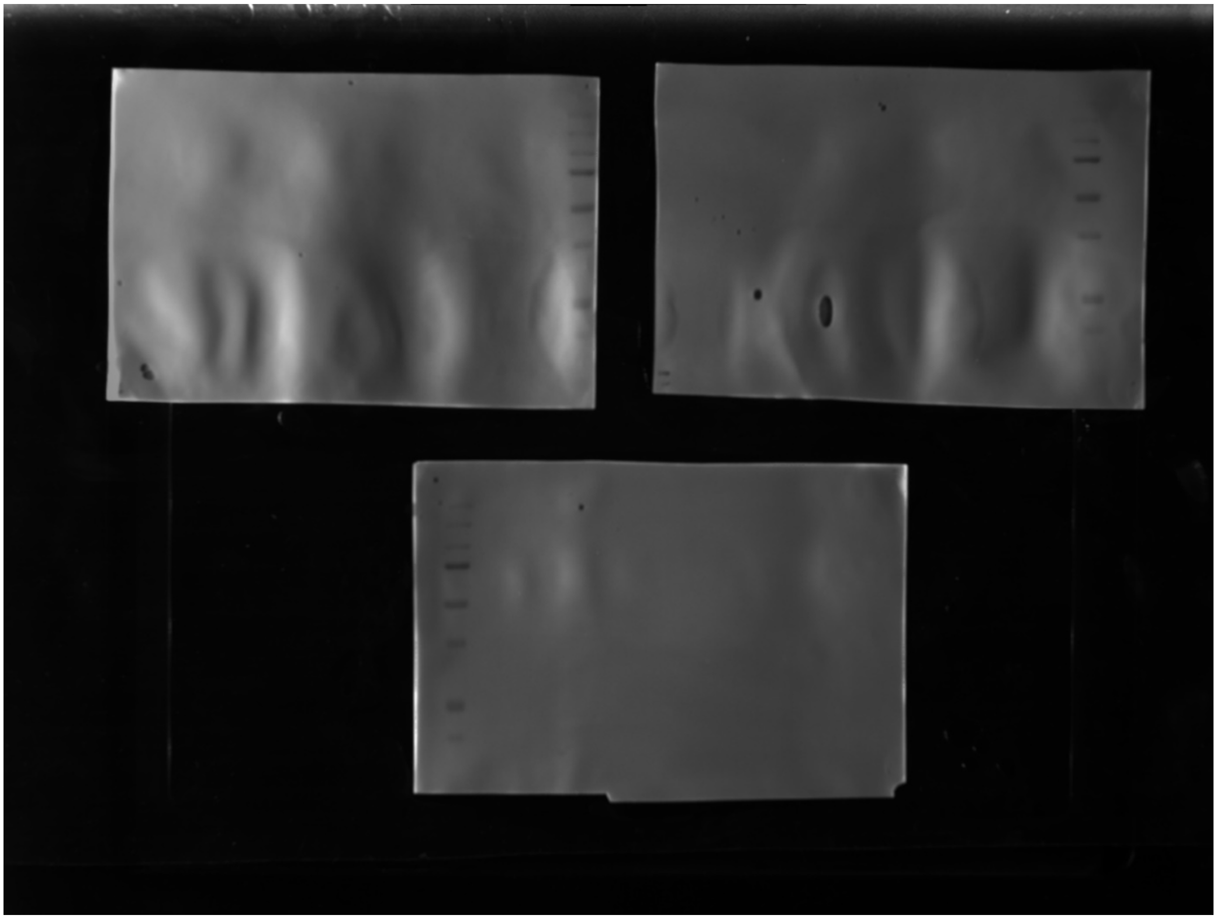

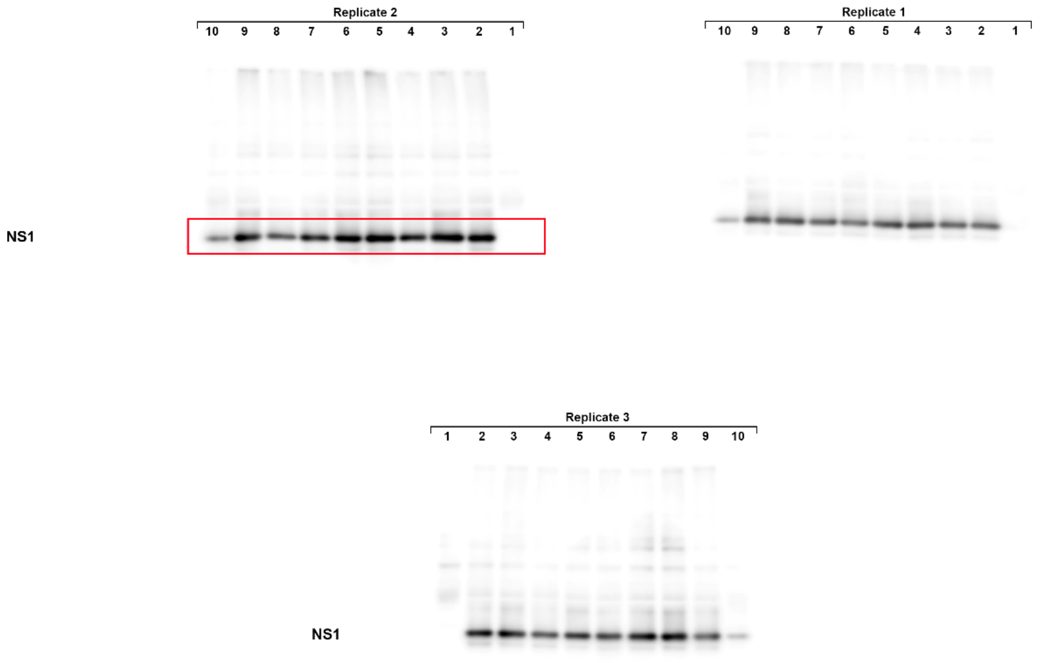

Full western blot membranes of NS1 protein captured by Azure400 (Rep 1-3), red boxes indicate bands presented in Fig 6; NS1 protein. Replicate 1-2 were flipped horizontally for publication.

1: Mock
2: Untreated cells with ZIKV infection
3: Treated cells with 0.1% DMSO before ZIKV infection for 6 hours
4: Treated cells with 0.1% DMSO before ZIKV infection for 3 hours
5: Treated cells with 0.1% DMSO before ZIKV infection for 1 hour
6: Treated cells with 0.1% DMSO during ZIKV infection
7: Treated cells with 100 µl of Baicalein before ZIKV infection for 6 hours
8: Treated cells with 100 µl of Baicalein before ZIKV infection for 3 hours
9: Treated cells with 100 µl of Baicalein before ZIKV infection for 1 hour
10: Treated cells with 100 µl of Baicalein during ZIKV infection

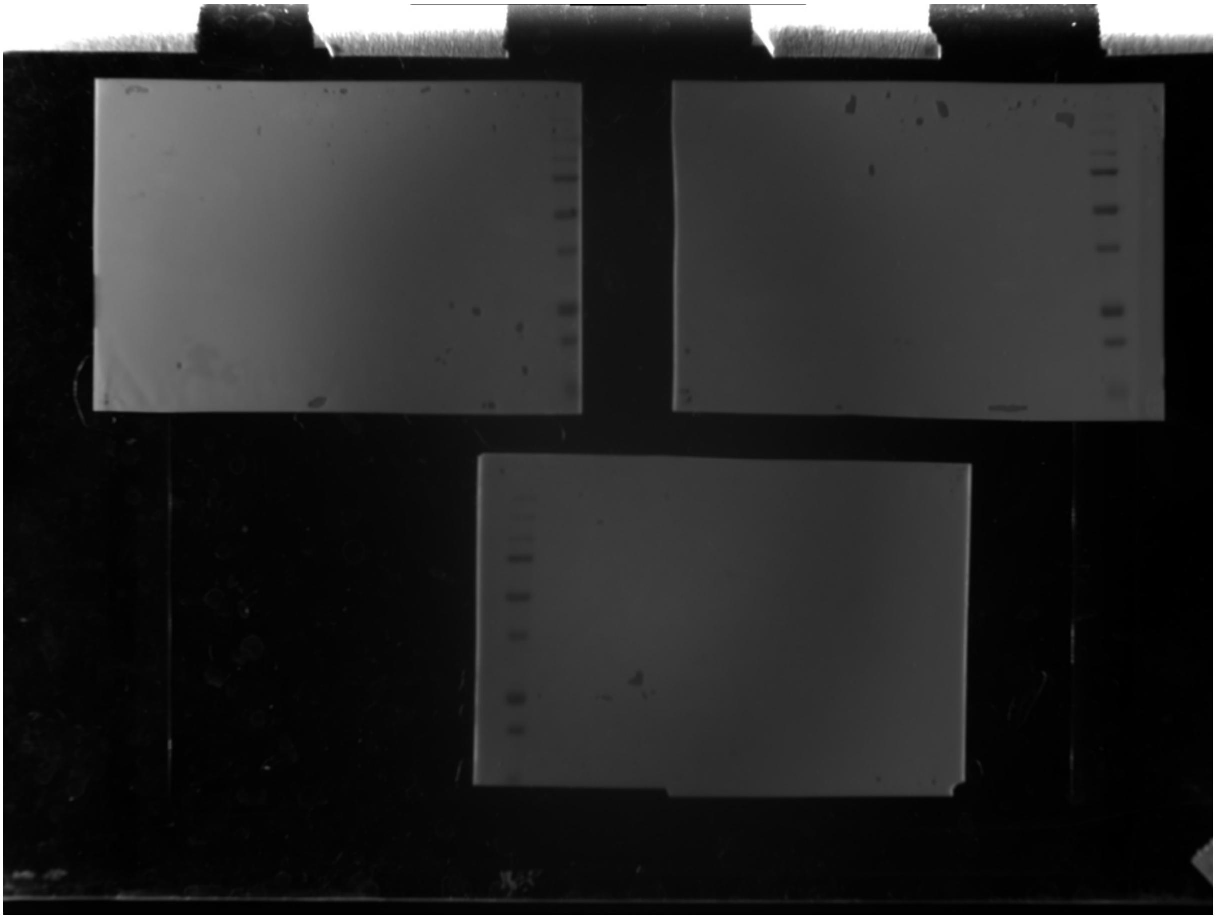

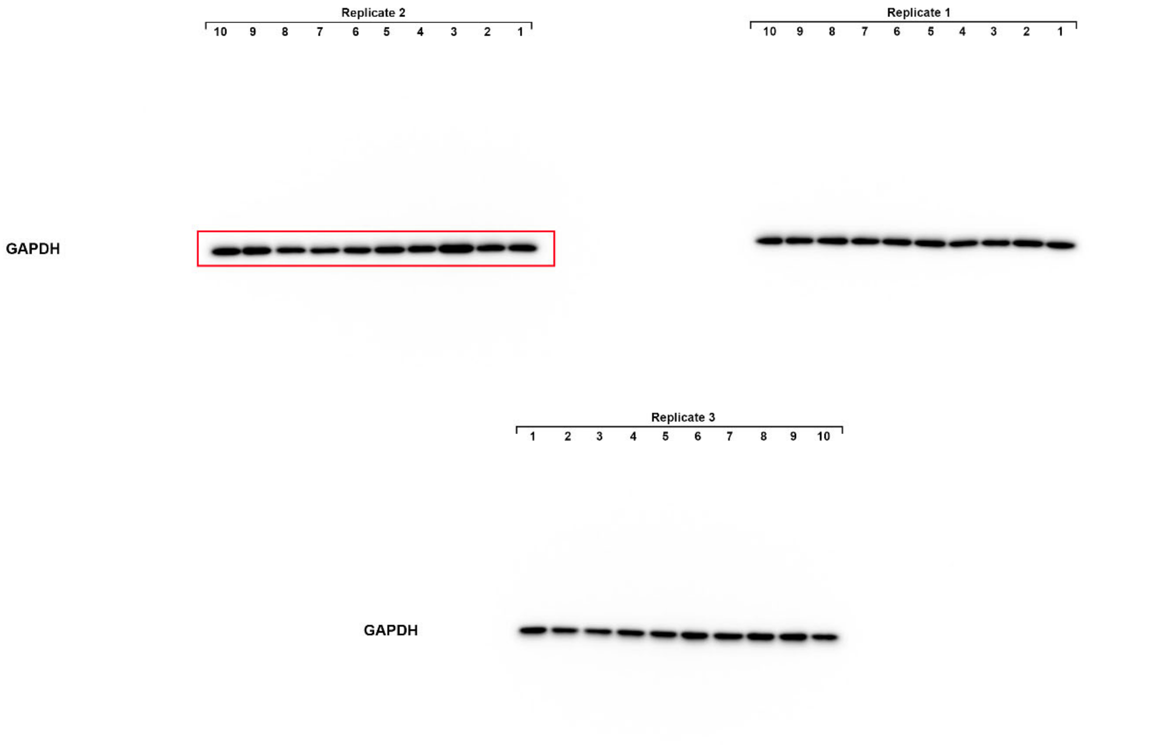

Full western blot membranes of GAPDH captured by Azure400 (Rep 1-3), red boxes indicate bands presented in Fig 6; GAPDH. Replicate 1-2 were flipped horizontally for publication.

1: Mock
2: Untreated cells with ZIKV infection
3: Treated cells with 0.1% DMSO before ZIKV infection for 6 hours
4: Treated cells with 0.1% DMSO before ZIKV infection for 3 hours
5: Treated cells with 0.1% DMSO before ZIKV infection for 1 hour
6: Treated cells with 0.1% DMSO during ZIKV infection
7: Treated cells with 100 µl of Baicalein before ZIKV infection for 6 hours
8: Treated cells with 100 µl of Baicalein before ZIKV infection for 3 hours
9: Treated cells with 100 µl of Baicalein before ZIKV infection for 1 hour
10: Treated cells with 100 µl of Baicalein during ZIKV infection

Western blot of viral protein expression at the post-adsorption stage

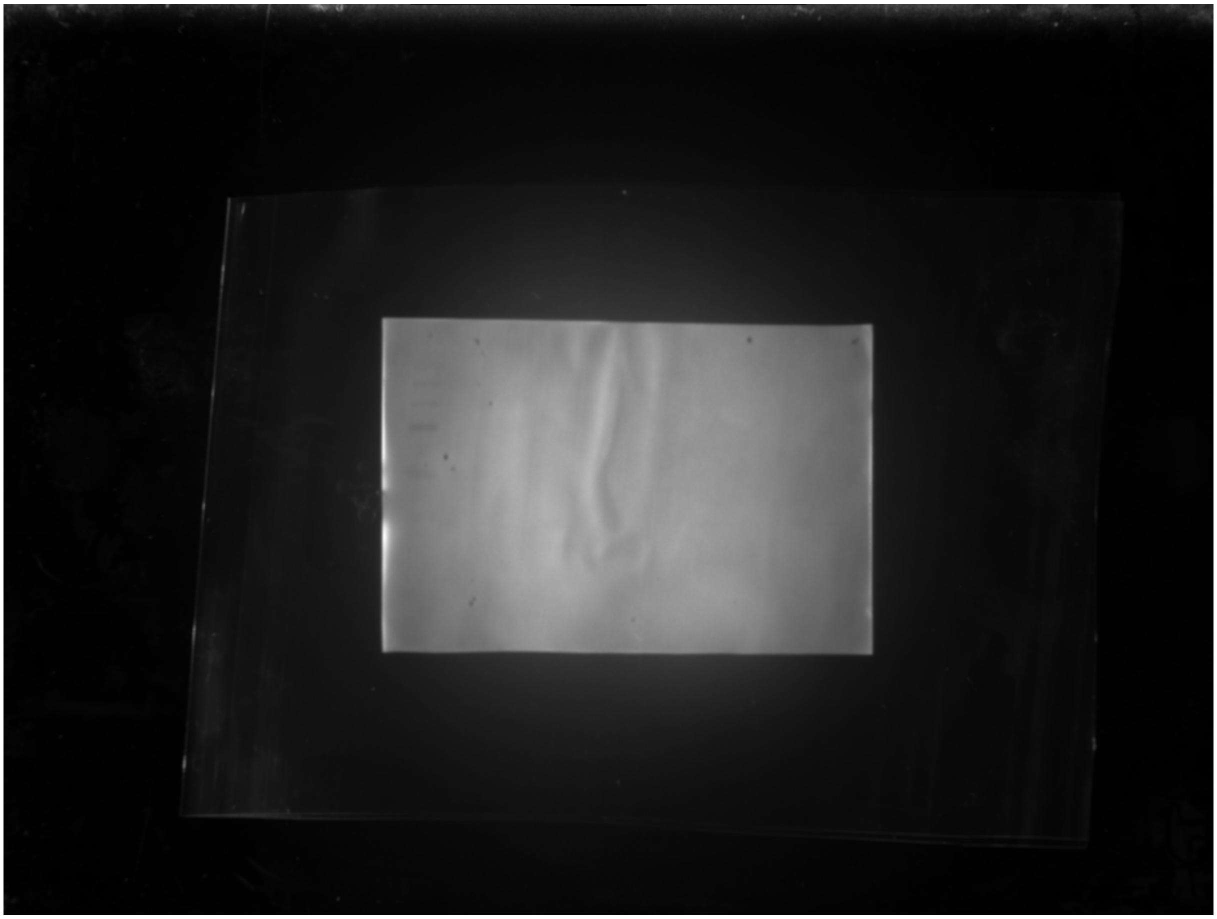

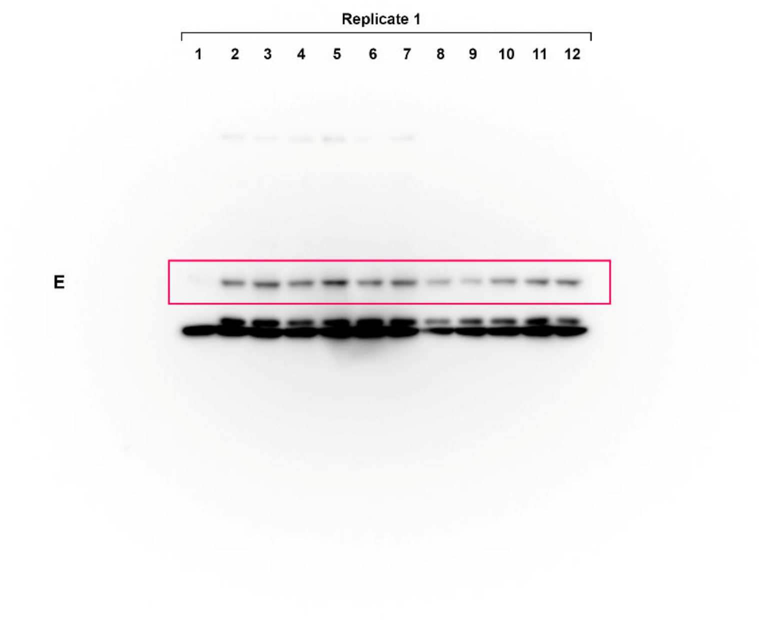

Full western blot membranes of E protein captured by Azure400 (Replicate 1), red boxes indicate bands presented in Fig 8; E protein.

1: Mock
2: Untreated cells with ZIKV infection
3: Treated cells with 0.1% DMSO after ZIKV infection for 0 hour
4: Treated cells with 0.1% DMSO after ZIKV infection for 1 hour
5: Treated cells with 0.1% DMSO after ZIKV infection for 3 hours
6: Treated cells with 0.1% DMSO after ZIKV infection for 6 hours
7: Treated cells with 0.1% DMSO after ZIKV infection for 9 hours
8: Treated cells with 100 µl of Baicalein after ZIKV infection for 0 hour
9: Treated cells with 100 µl of Baicalein after ZIKV infection for 1 hour
10: Treated cells with 100 µl of Baicalein after ZIKV infection for 3 hours
11: Treated cells with 100 µl of Baicalein after ZIKV infection for 6 hours
12: Treated cells with 100 µl of Baicalein after ZIKV infection for 9 hours

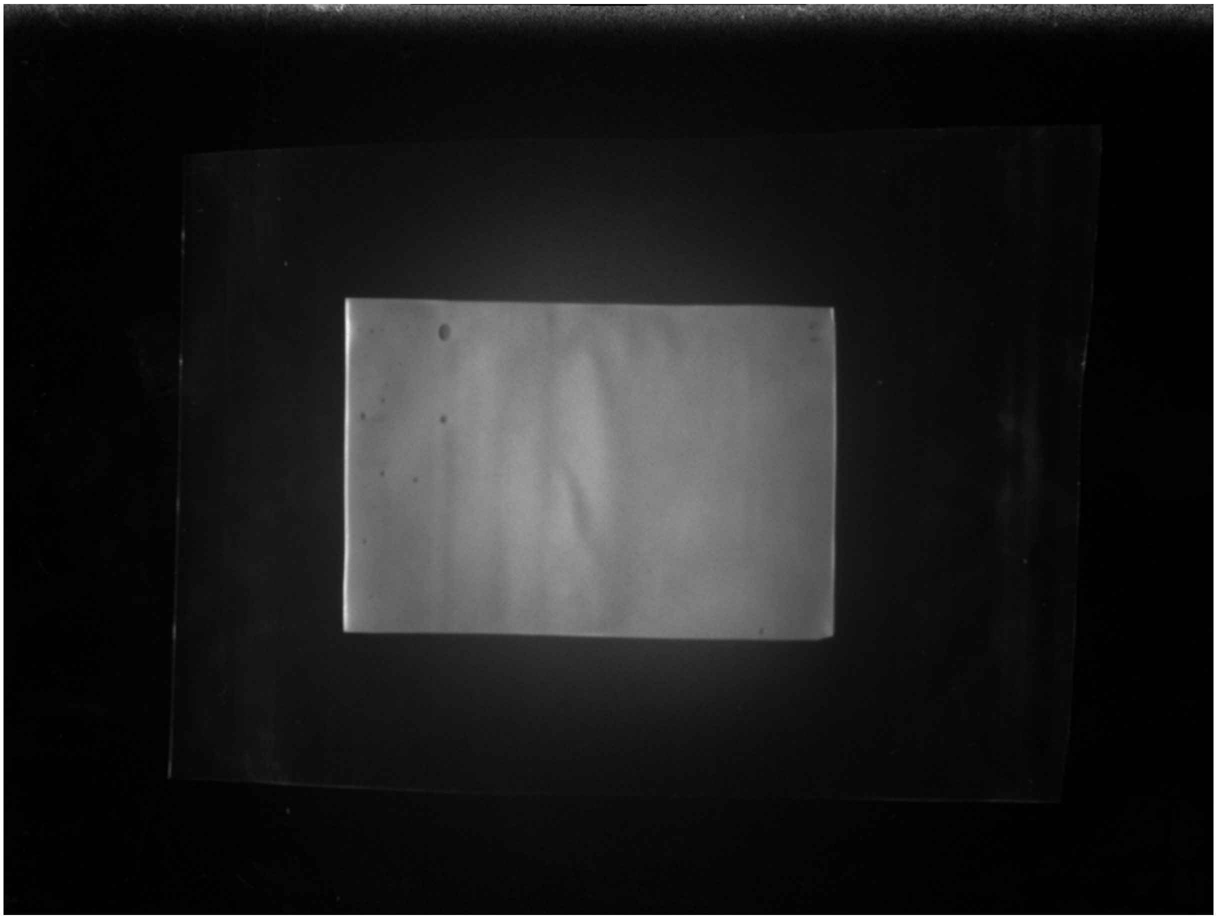

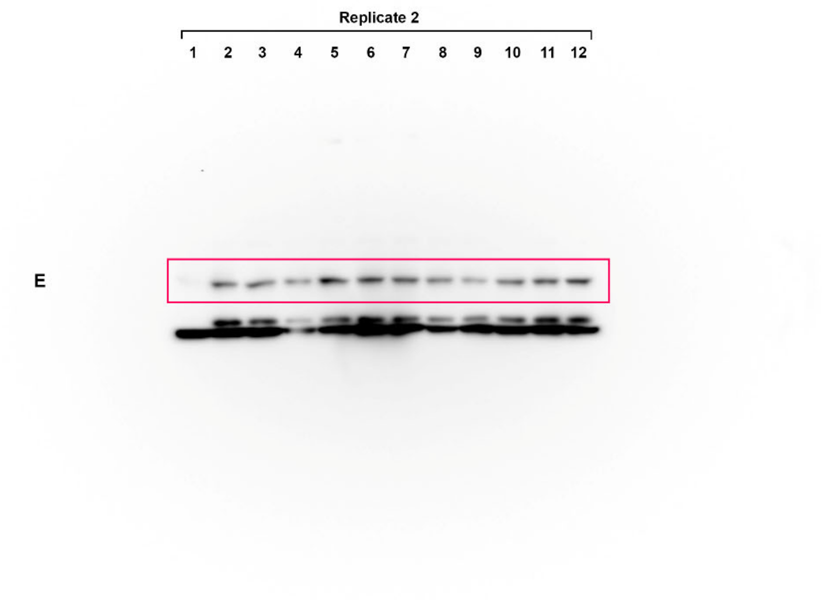

Full western blot membranes of E protein captured by Azure400 (Replicate 2), red boxes indicate bands presented in Fig 8; E protein.

1: Mock
2: Untreated cells with ZIKV infection
3: Treated cells with 0.1% DMSO after ZIKV infection for 0 hour
4: Treated cells with 0.1% DMSO after ZIKV infection for 1 hour
5: Treated cells with 0.1% DMSO after ZIKV infection for 3 hours
6: Treated cells with 0.1% DMSO after ZIKV infection for 6 hours
7: Treated cells with 0.1% DMSO after ZIKV infection for 9 hours
8: Treated cells with 100 µl of Baicalein after ZIKV infection for 0 hour
9: Treated cells with 100 µl of Baicalein after ZIKV infection for 1 hour
10: Treated cells with 100 µl of Baicalein after ZIKV infection for 3 hours
11: Treated cells with 100 µl of Baicalein after ZIKV infection for 6 hours
12: Treated cells with 100 µl of Baicalein after ZIKV infection for 9 hours

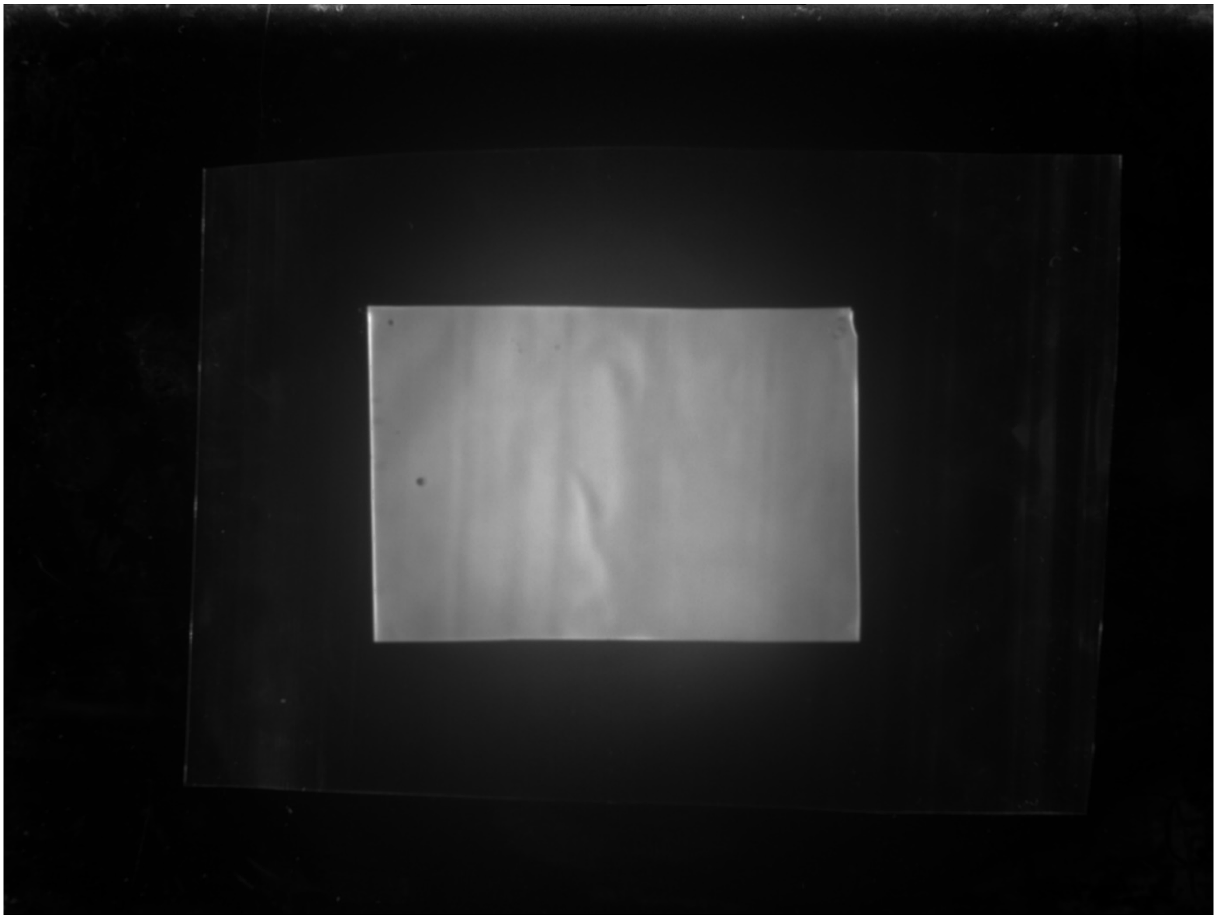

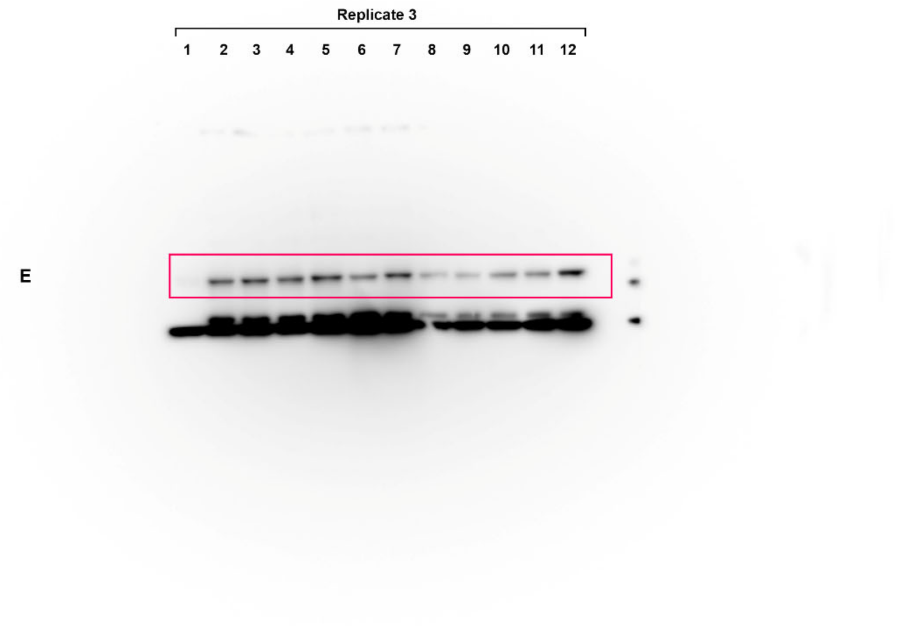

Full western blot membranes of E protein captured by Azure400 (Replicate 3), red boxes indicate bands presented in Fig 8; E protein.

1: Mock
2: Untreated cells with ZIKV infection
3: Treated cells with 0.1% DMSO after ZIKV infection for 0 hour
4: Treated cells with 0.1% DMSO after ZIKV infection for 1 hour
5: Treated cells with 0.1% DMSO after ZIKV infection for 3 hours
6: Treated cells with 0.1% DMSO after ZIKV infection for 6 hours
7: Treated cells with 0.1% DMSO after ZIKV infection for 9 hours
8: Treated cells with 100 µl of Baicalein after ZIKV infection for 0 hour
9: Treated cells with 100 µl of Baicalein after ZIKV infection for 1 hour
10: Treated cells with 100 µl of Baicalein after ZIKV infection for 3 hours
11: Treated cells with 100 µl of Baicalein after ZIKV infection for 6 hours
12: Treated cells with 100 µl of Baicalein after ZIKV infection for 9 hours

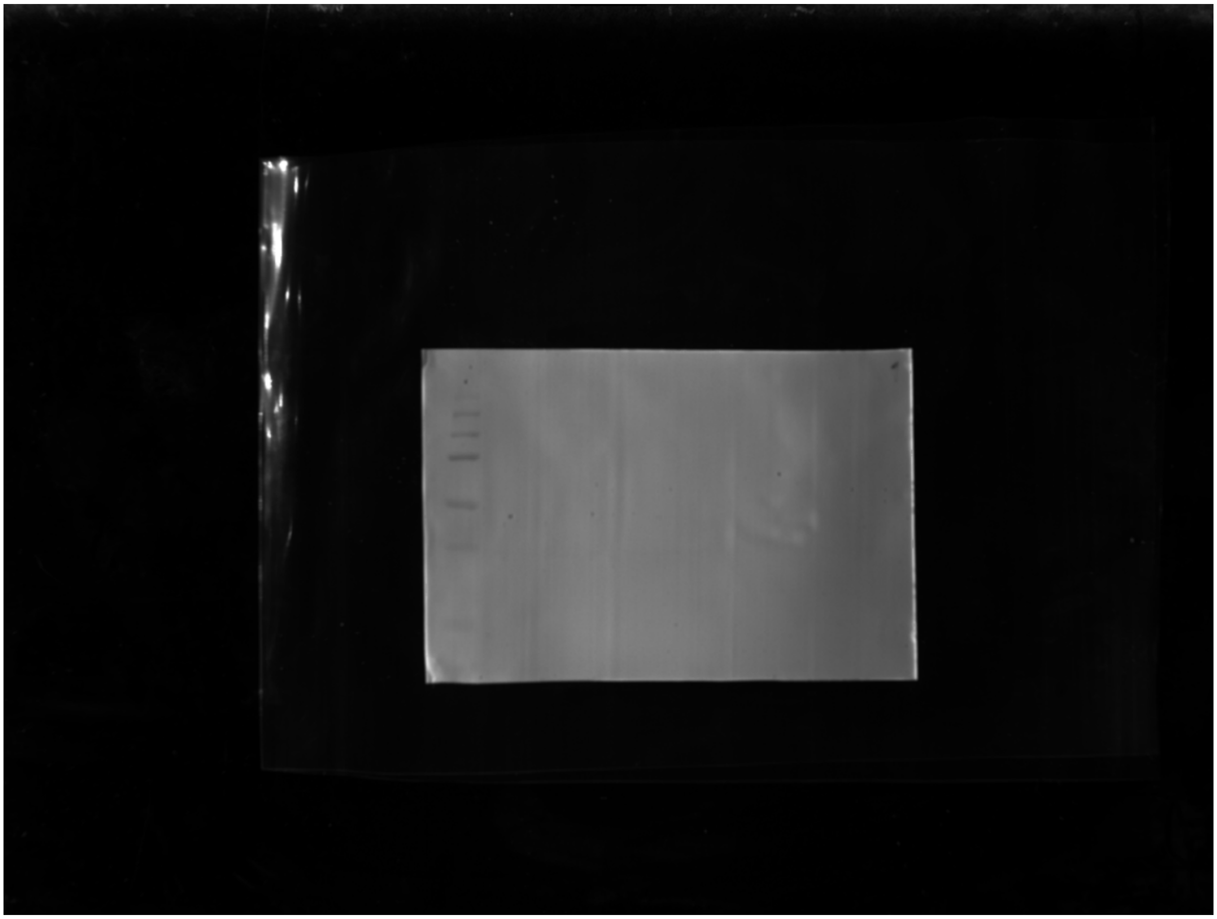

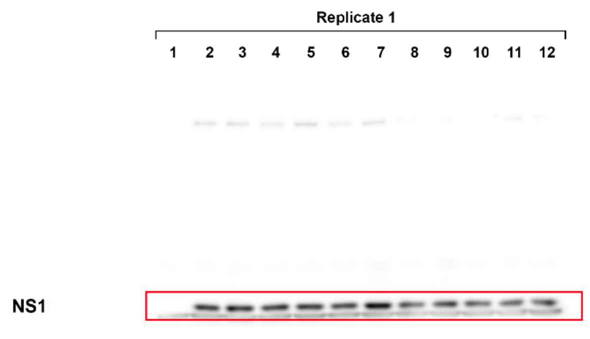

Full western blot membranes of NS1 protein captured by Azure400 (Rep 1), red boxes indicate bands presented in Fig 8; NS1 protein.

1: Mock
2: Untreated cells with ZIKV infection
3: Treated cells with 0.1% DMSO after ZIKV infection for 0 hour
4: Treated cells with 0.1% DMSO after ZIKV infection for 1 hour
5: Treated cells with 0.1% DMSO after ZIKV infection for 3 hours
6: Treated cells with 0.1% DMSO after ZIKV infection for 6 hours
7: Treated cells with 0.1% DMSO after ZIKV infection for 9 hours
8: Treated cells with 100 µl of Baicalein after ZIKV infection for 0 hour
9: Treated cells with 100 µl of Baicalein after ZIKV infection for 1 hour
10: Treated cells with 100 µl of Baicalein after ZIKV infection for 3 hours
11: Treated cells with 100 µl of Baicalein after ZIKV infection for 6 hours
12: Treated cells with 100 µl of Baicalein after ZIKV infection for 9 hours

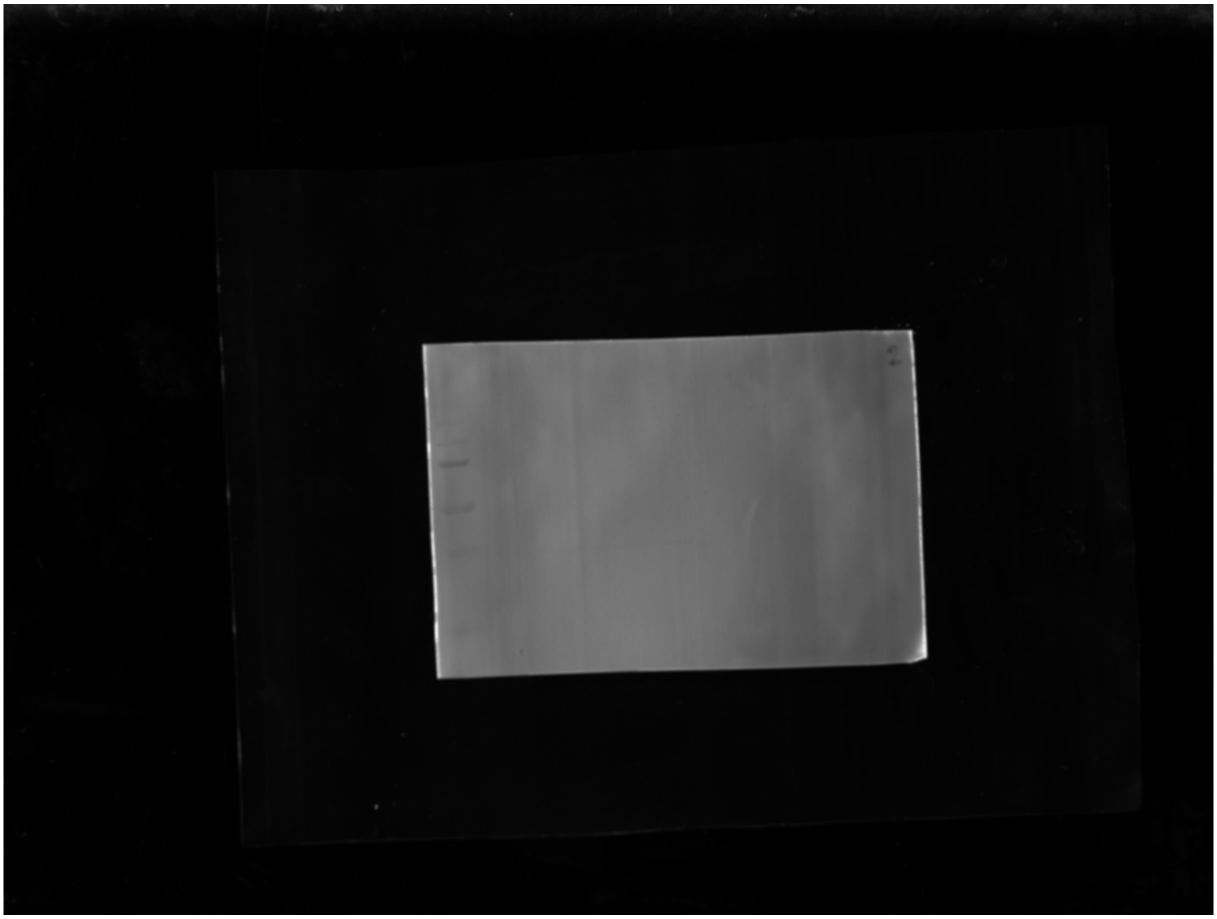

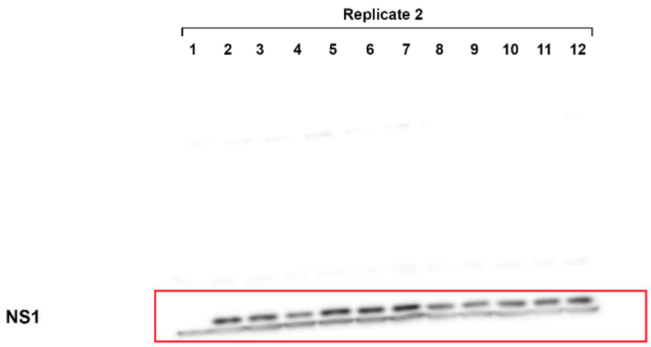

Full western blot membranes of NS1 protein captured by Azure400 (Rep 2), red boxes indicate bands presented in Fig 8; NS1 protein.

1: Mock
2: Untreated cells with ZIKV infection
3: Treated cells with 0.1% DMSO after ZIKV infection for 0 hour
4: Treated cells with 0.1% DMSO after ZIKV infection for 1 hour
5: Treated cells with 0.1% DMSO after ZIKV infection for 3 hours
6: Treated cells with 0.1% DMSO after ZIKV infection for 6 hours
7: Treated cells with 0.1% DMSO after ZIKV infection for 9 hours
8: Treated cells with 100 µl of Baicalein after ZIKV infection for 0 hour
9: Treated cells with 100 µl of Baicalein after ZIKV infection for 1 hour
10: Treated cells with 100 µl of Baicalein after ZIKV infection for 3 hours
11: Treated cells with 100 µl of Baicalein after ZIKV infection for 6 hours
12: Treated cells with 100 µl of Baicalein after ZIKV infection for 9 hours

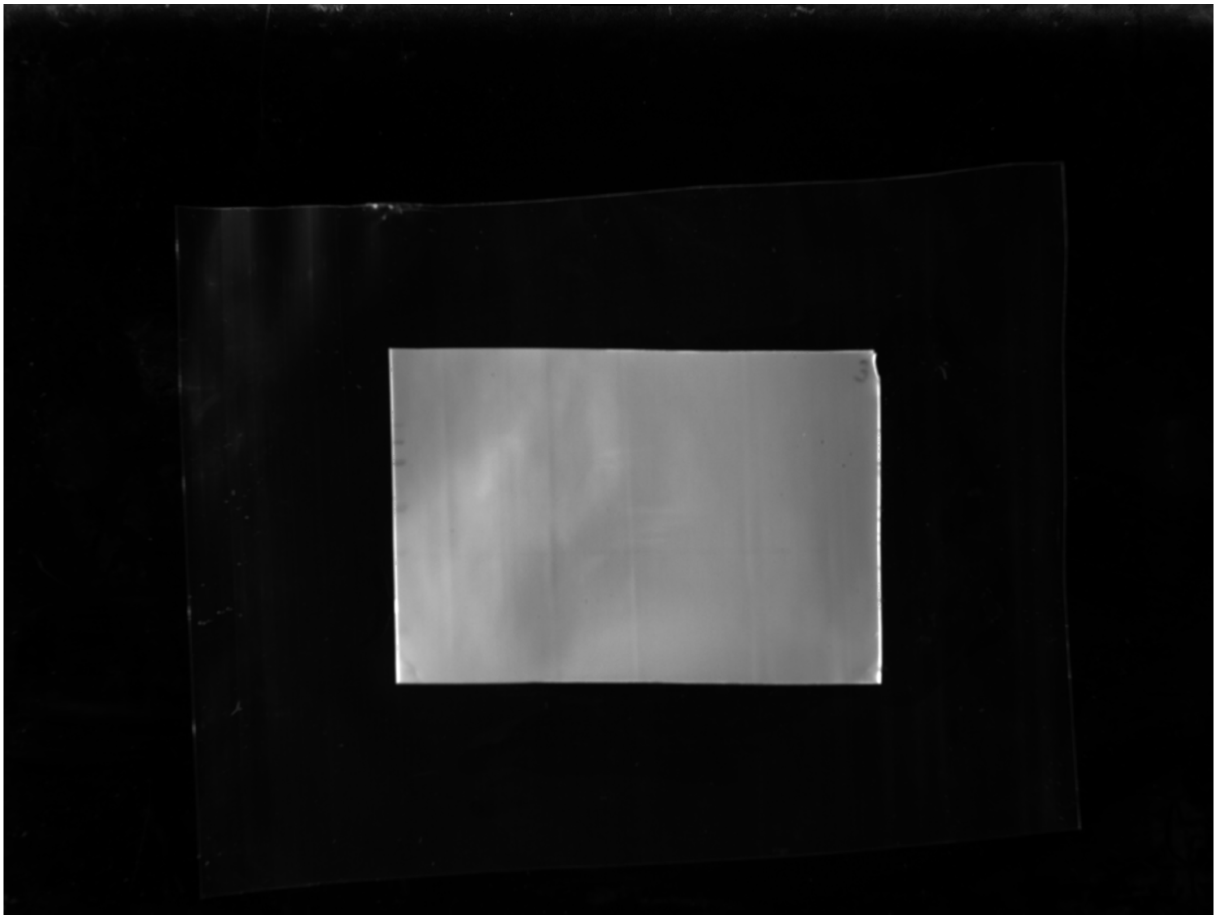

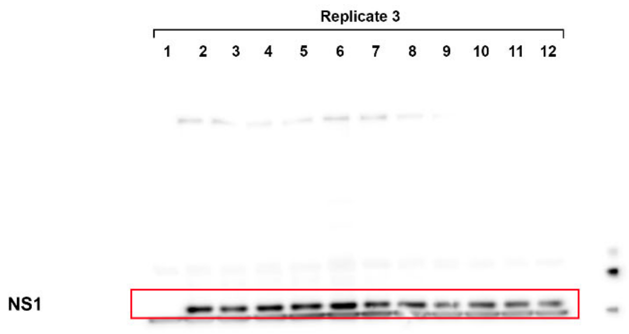

Full western blot membranes of NS1 protein captured by Azure400 (Rep 3), red boxes indicate bands presented in Fig 8; NS1 protein.

1: Mock
2: Untreated cells with ZIKV infection
3: Treated cells with 0.1% DMSO after ZIKV infection for 0 hour
4: Treated cells with 0.1% DMSO after ZIKV infection for 1 hour
5: Treated cells with 0.1% DMSO after ZIKV infection for 3 hours
6: Treated cells with 0.1% DMSO after ZIKV infection for 6 hours
7: Treated cells with 0.1% DMSO after ZIKV infection for 9 hours
8: Treated cells with 100 µl of Baicalein after ZIKV infection for 0 hour
9: Treated cells with 100 µl of Baicalein after ZIKV infection for 1 hour
10: Treated cells with 100 µl of Baicalein after ZIKV infection for 3 hours
11: Treated cells with 100 µl of Baicalein after ZIKV infection for 6 hours
12: Treated cells with 100 µl of Baicalein after ZIKV infection for 9 hours

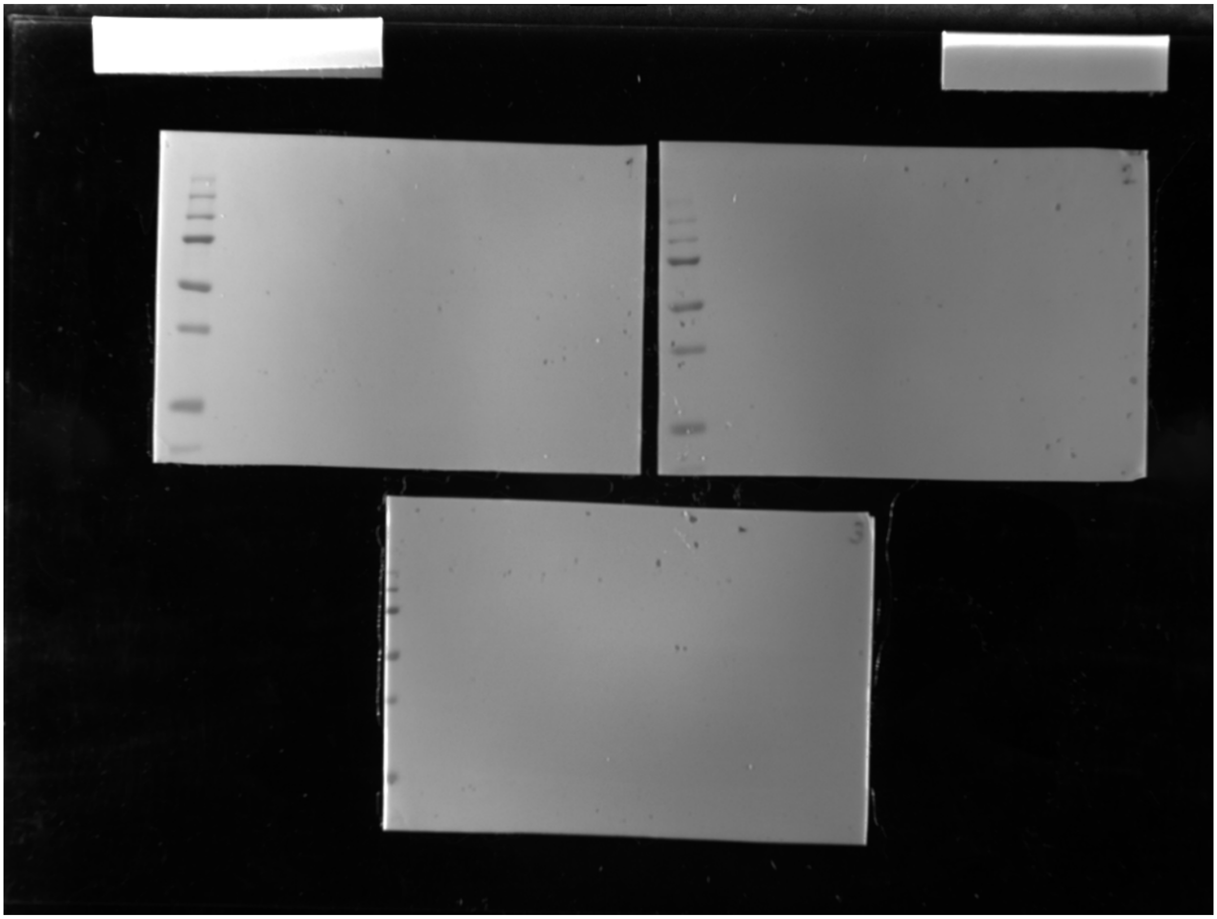

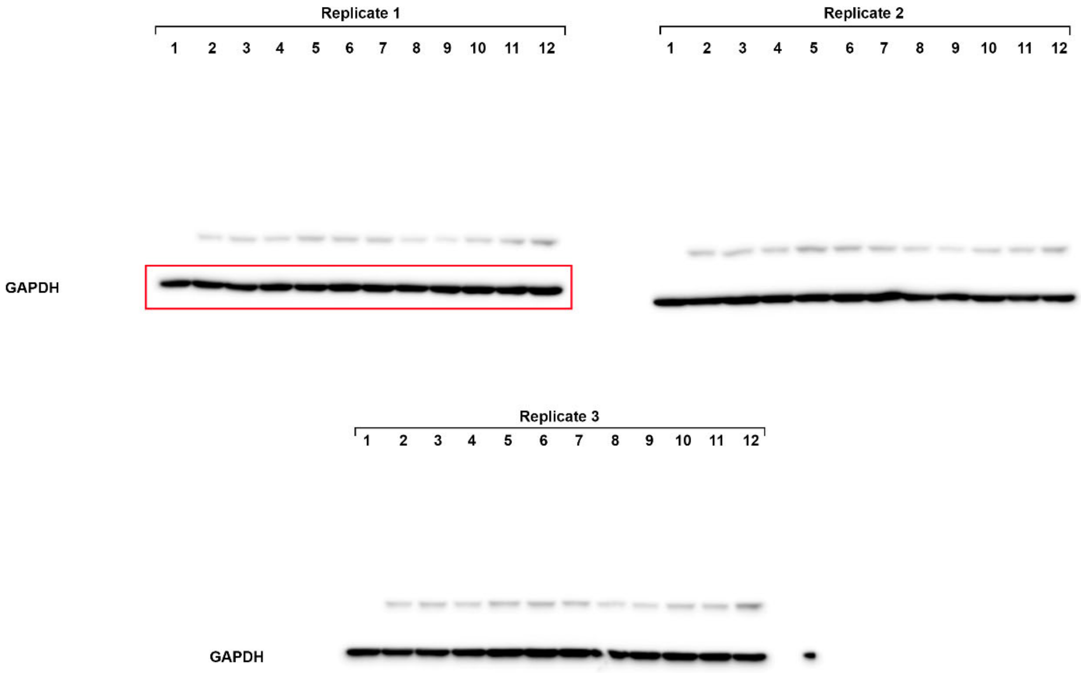

Full western blot membranes of GAPDH captured by Azure400 (Rep 1-3), red boxes indicate bands presented in Fig 8; GAPDH.

1: Mock
2: Untreated cells with ZIKV infection
3: Treated cells with 0.1% DMSO after ZIKV infection for 0 hour
4: Treated cells with 0.1% DMSO after ZIKV infection for 1 hour
5: Treated cells with 0.1% DMSO after ZIKV infection for 3 hours
6: Treated cells with 0.1% DMSO after ZIKV infection for 6 hours
7: Treated cells with 0.1% DMSO after ZIKV infection for 9 hours
8: Treated cells with 100 µl of Baicalein after ZIKV infection for 0 hour
9: Treated cells with 100 µl of Baicalein after ZIKV infection for 1 hour
10: Treated cells with 100 µl of Baicalein after ZIKV infection for 3 hours
11: Treated cells with 100 µl of Baicalein after ZIKV infection for 6 hours
12: Treated cells with 100 µl of Baicalein after ZIKV infection for 9 hours
